# Molecular detection of exosomal miRNAs of blood serum for prognosis of colorectal cancer

**DOI:** 10.1038/s41598-024-58536-3

**Published:** 2024-04-17

**Authors:** Tahani Bakhsh, Safiah Alhazmi, Ali Farsi, Abdulaziz S. Yusuf, Amani Alharthi, Safa H. Qahl, Maha Ali Alghamdi, Faisal A. Alzahrani, Ola H. Elgaddar, Mohanad A. Ibrahim, Ahmed Bahieldin

**Affiliations:** 1 Department of Biology, College of Science, University of Jeddah, Jeddah, Saudi Arabia; 2 Department of Biological Sciences, Faculty of Science, King Abdulaziz University, 21589 Jeddah, Saudi Arabia; 3 Department of Surgery, Faculty of Medicine, King Abdulaziz University, 21589 Jeddah, Saudi Arabia; 4 Department of Biochemistry, Faculty of science, Stem Cell Unit, King Fahad Center for Medical Research, King Abdulaziz University, 21589 Jeddah, Saudi Arabia; 5 Department of Biology, College of Science Al-Zulfi, Majmaah University, 11952 Majmaah, Saudi Arabia; 6 Department of Biotechnology, College of Science, Taif University, Taif, Saudi Arabia; 7 Department of Chemical Pathology, Alexandria University, Alexandria, Egypt; 8 Data Science Program, King Abdullah International Medical Research Center, 11481 Riyadh, Saudi Arabia; 9 Medical Laboratory Sciences Department, Fakeeh College for Medical Sciences, 21461 Jeddah, Saudi Arabia; 10 Immunology Unit, King Fahad Medical Research Centre, King Abdulaziz University, 80200 Jedaah, Saudi Arabia; 11 Neuroscience and Geroscience Research Unit, King Fahad Medical Research Centre, King Abdulaziz University, 80200 Jeddah, Saudi Arabia; 12 Central lab of biological Sciences, Faculty of Sciences, King Abdulaziz University, 80200 Jeddah, Saudi Arabia

**Keywords:** Exosomes, miRNAs, Colorectal cancer, Biomarkers, Deep sequencing, Biochemistry, Biological techniques, Biotechnology, Cancer, Chemical biology, Computational biology and bioinformatics, Genetics, Molecular biology, Biomarkers, Diseases, Gastroenterology, Health care, Medical research, Molecular medicine, Oncology, Pathogenesis, Risk factors

## Abstract

Colorectal cancer (CRC) is the third most common cancer affecting people. The discovery of new, non-invasive, specific, and sensitive molecular biomarkers for CRC may assist in the diagnosis and support therapeutic decision making. Exosomal miRNAs have been demonstrated in carcinogenesis and CRC development, which makes these miRNAs strong biomarkers for CRC. Deep sequencing allows a robust high-throughput informatics investigation of the types and abundance of exosomal miRNAs. Thus, exosomal miRNAs can be efficiently examined as diagnostic biomarkers for disease screening. In the present study, a number of 660 mature miRNAs were detected in patients diagnosed with CRC at different stages. Of which, 29 miRNAs were differentially expressed in CRC patients compared with healthy controls. Twenty-nine miRNAs with high abundance levels were further selected for subsequent analysis. These miRNAs were either highly up-regulated (e.g., let-7a-5p, let-7c-5p, let-7f-5p, let-7d-3p, miR-423-5p, miR-3184-5p, and miR-584) or down-regulated (e.g., miR-30a-5p, miR-99-5p, miR-150-5p, miR-26-5p and miR-204-5p). These miRNAs influence critical genes in CRC, leading to either tumor growth or suppression. Most of the reported diagnostic exosomal miRNAs were shown to be circulating in blood serum. The latter is a novel miRNA that was found in exosomal profile of blood serum. Some of the predicted target genes of highly expressed miRNAs participate in several cancer pathways, including CRC pathway. These target genes include tumor suppressor genes, oncogenes and DNA repair genes. Main focus was given to multiple critical signaling cross-talking pathways including transforming growth factor β (TGFβ) signaling pathways that are directly linked to CRC. In conclusion, we recommend further analysis in order to experimentally confirm exact relationships between selected differentially expressed miRNAs and their predicted target genes and downstream functional consequences.

## Introduction

Colorectal cancer (CRC) is result of the proliferation of cancerous cells from the mucosal layer in the colon and rectum^1^. CRC is a major public health concern in the world, as it is the third most common cause of cancer death^2^. Untreated CRC eventually progresses to systemic metastasis, which is the major cause of CRC mortality^3^. When first diagnosed, the stage of each individual patient’s disease needs to established, as it is the most important step in setting a treatment plan(see file for reference). Earlier detection of CRC and more accurate assessment of the cancer stage could potentially help physicians better tailor cancer therapy to the patients’ exact stage. Many risk factors contribute to the development of CRC, and individuals' poor lifestyles may also contribute to rise in number of CRC cases^4^. It was demonstrated that several tumor suppressor genes in CRC such as *APC*, *TGFβR2*, *SMAD4*, *PTEN* and *TP53*, were inactivated, while in contrast, oncogenes such as *KRAS*, *BRAF*, *β-catenin*, and *MYC* were overexpressed^5–7^. It was exhibited that 20–30% of CRC instances are caused by hereditary mutations, while the remaining cases of CRCs are sporadic in nature^8^.

MicroRNAs (miRNAs) are short non-coding RNAs around 18–23 nucleotides (nt) that regulate transcription and post-transcriptional modifications of their target genes^9^. Roles of miRNAs include inhibition or degradation of mRNA during binding to mRNAs in 3′-UTR termini^10^. Although most physiological functions of miRNAs are undefined, their expression profiles have been illustrated in various pathology fields^11^. Moreover, sensitivity and simplicity of sampling miRNAs contribute to production of appropriate non-invasive biomarkers^11^. Spotting properties of miRNAs could assist in comprehending carcinogenesis machinery, which could potentially lead to them becoming biomarkers that assist in establishing CRC diagnosis and prognosis^12^. However, biology of miRNAs is a massive area that is required for studying and understanding their mechanisms and applications in clinical cancer diagnosis and treatment.

Because exosomes play a critical role in transferring or exchanging materials between distant tissues and mediating intercellular communication^13,14^, they have been employed in many studies to assess their role as diagnostic and prognostic biomarkers for CRC^15^. Exosomes have been distinguished as significant transporters for miRNAs in serum fields^16^. Expression of serum miRNA is noted to be different in CRC patients, which needs more examination to identify link between expression of specific miRNAs in both serum and tissues of CRC patients. Because of insignificance of miRNA techniques, exosomal miRNAs might be conceivably utilized as indicative of biomarkers for different types of cancer^13^. Study of serum exosomal miR-92a-3p and miR-17-5p for diagnosis of primary and metastatic CRC suggested that up-regulation of these miRNAs directly correlated with stages of CRC^17^. Another study identified that low expression of circulating exosomal miR-4772-3p, isolated from serum, had been considered a predictive biomarker for cancer recurrence in colon cancer patients at stages II and III^18^. Next-generation sequencing (NGS) can provide a robust informatic analysis related to sorts and abundance of exosomal miRNAs. Thus, exosomal miRNAs can be efficiently examined as diagnostic biomarkers for several diseases^19^.

*TGFβ* gene family are normally regulated in epithelial cells, however, dysregulation of any member of this family can contribute to increasing tumorigenesis. Canonical TGFβ signaling is regulated by TGFβR1, and TGFβR2, which phosphorylate SMAD2 and SMAD3, after binding to TGFβ ligands. Then, activated SMAD2 and SMAD3 comprise a complex with SMAD4. SMAD complex is a nucleoprotein that modulates expression of several target genes and affects tumor growth by controlling cell proliferation, apoptosis, metastasis, and angiogenesis (Massagué et al*.*^20^; Feng and Derynck^21^). miRNAs have been shown to influence formation and activation of TGFβ signaling pathway, and miRNAs/TGF interaction acts in occurrence of various malignancies, including gastric, breast, lung, pancreatic, rectal and colon cancers (Soleimani et al*.*^22,23^).

In this study, deep sequencing approach was utilized for total blood RNAs to comparatively analyze circulating exosomal miRNAs expression in CRC patients at different cancer stages. The most strongly up-regulated exosomal miRNAs (e.g., let-7a-5p, let-7c-5p, let-7f.-5p, let-7d-3p, miR-423-5p, miR-3184-5p, and miR-584-5p) or down-regulated (e.g., miR-30a-5p, miR-99-5p, miR-150-5p, miR-26-5p, and miR-204-5p) were further investigated. These highly expressed miRNAs were predicted to target genes in cancer pathways, including CRC pathway. These target genes involve tumor-suppressor genes, oncogenes, and DNA repair genes. Many important signaling pathways such as transforming growth factors (TGFβ) were shown to directly associate with CRC. This work could support clarifying role of exosomal miRNAs in regulating expression of target genes that contribute to development of CRC progression. Exosomal miRNAs may, thus, distinguish CRC cases from healthy controls and serve as biomarkers for CRC diagnosis and prognosis.

## Results

### Morphological features of isolated exosomes

**Figure 1:**
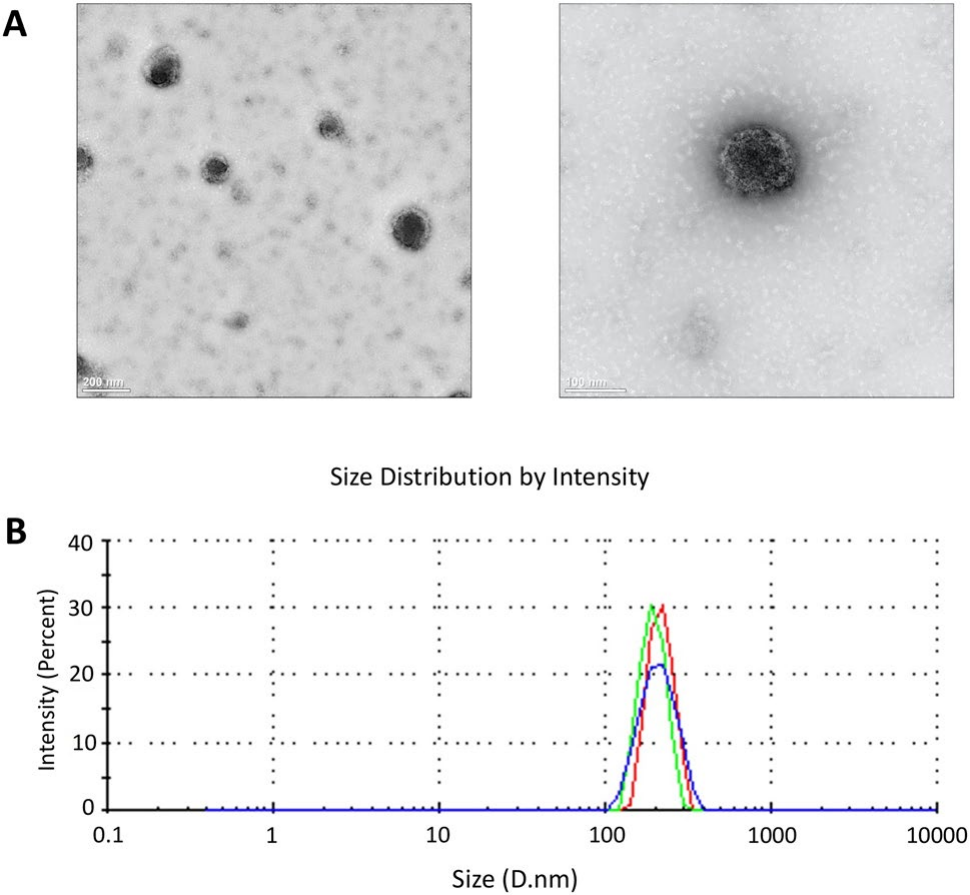
Characteristics of serum-derived exosomes from CRC patients. (**A**) TEM presented exosome morphology with scale bars are 200 nm (left row) and 100 nm (right row). (**B**) Physical properties of exosomes show size distribution of serum-derived EVs including exosomes, as assessed by Zetasizer Nano. The curve depicts means, and standard deviations (SD) from three experiment replicates that yielded similar results.

Exosome pellets obtained from membrane using spin column were examined firstly by TEM to prove isolation of exosomes on a morphological level of which they were shown as sphere-shaped extracellular vesicles harboring typical bilayer and their sizes were estimated to be between 100–200 nm (Fig. 1A). In addition, Zetasizer Nano (ZN) was used to detect physical properties of exosomes and size distribution of serum-derived extracellular vesicles (EVs) including exosomes with three replicates of a typical experiment, and similar findings were reached, where Z-Average was 232.1d.nm with Pdl of 0.157 indicating that most exosomes were collected with other additional EVs during extraction (Fig. 1B). These findings demonstrated that exosomes have been effectively isolated from serum, then, they were used further to detect new serum exosomal biomarkers for CRC.

### Exosomal miRNAs expression profiles

**Figure 2:**
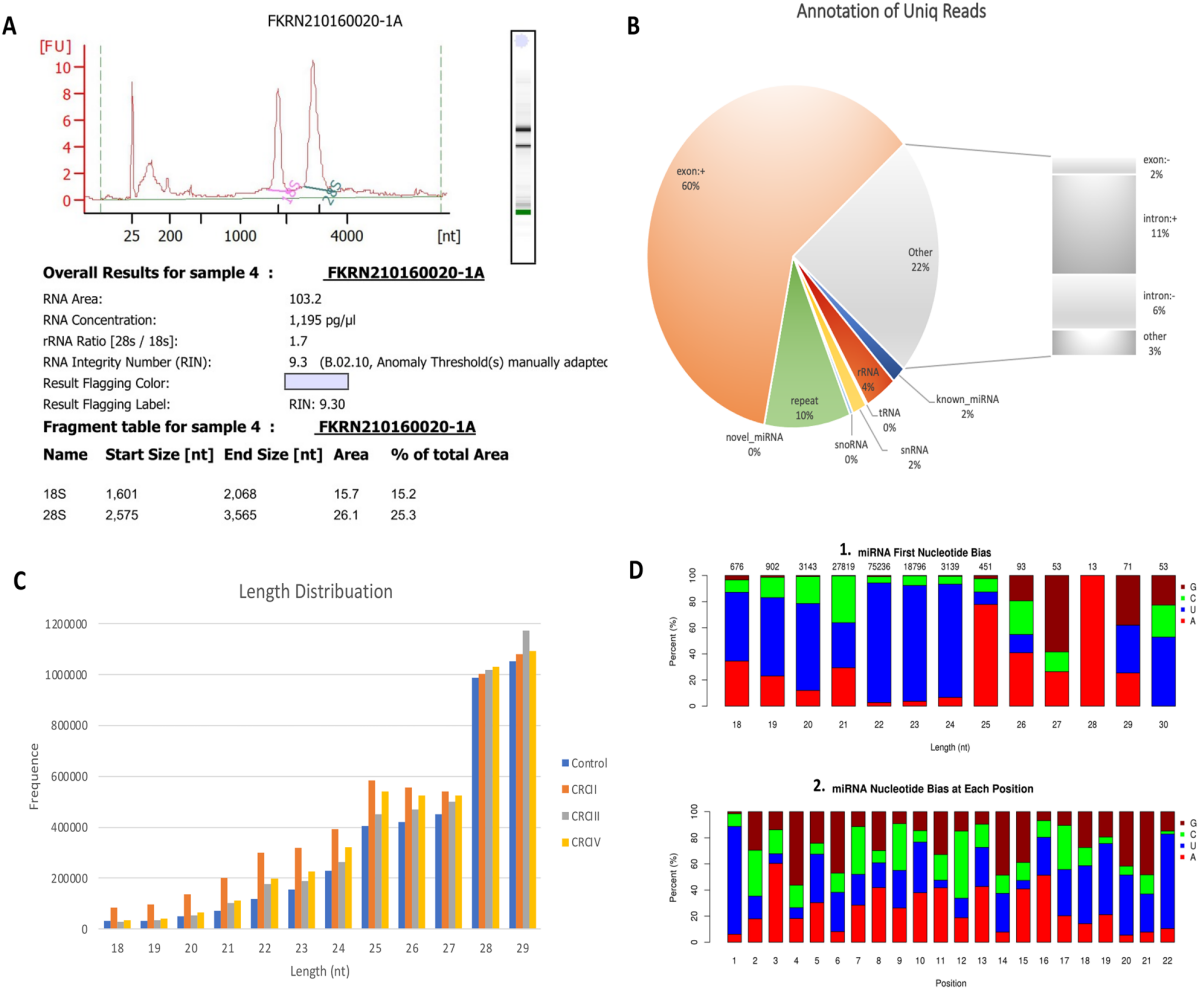
Overview of exosomal miRNAs analysis. (**A**) RNA integrity number (RIN) for total RNA (Agilent 2100 Bioanalyzer). (**B**) Pie chart showing summary of annotation of total RNA unique reads with percentages of several RNA classes detected in dataset. (**C**) Length distribution of exosomal miRNAs sequences in control and CRC groups (II, III and IV) library. (**D**) First nucleotide bias in resulted miRNAs with 18–30 nt length **(1)** and bias at different positions of miRNAs with 22 nt **(2)**.

Small amounts of RNA were taken before approaching RNA sequencing using Agilent® 2100 Bioanalyzer (Agilent Technologies, United States) to test RNA integrity number (RIN), and results demonstrated high integrity of RNA (9.30) (Fig. 2A). Total RNA samples were taken from nine CRC patients at different stages (CRCII, CRCIII & CRCIV) and three healthy controls. miRNA sequencing libraries yielded 12,002,536, 12,850,941, 13,830,980 and 12,475,000 raw reads from control, CRCII, CRCIII, and CRCIV, respectively. Number of clean reads in libraries of control, CRCII, CRCIII and CRCIV were 11,798,483 (98.20%), 12,658,663 (98.48%), 13,641,029 (98.64%), and 12,316,277 (98.69%), respectively. miRNA annotation presented known miRNAs, rRNA, tRNAs, small nuclear (snRNA), small nucleolar (snoRNA), repeats, novel miRNAs, exonic RNAs, and intronic RNAs in serum-derived exosomes. Expectedly, most common reads in annotation referred to exon (60.50%) followed by others (22%), repeats (10%), rRNA (4%), known miRNAs (2%), snRNA (2%), snoRNA (0.35), tRNAs (0.20%), while the least was for novel miRNAs (0.01) (Fig. 2B & S1). Length distribution of clean reads of pre-miRNAs in control, CRCII, CRCIII, and CRCIV libraries were between 18–29 nt (longer reads were removed), while length of mature miRNA was between 21–22 nt (Fig. 2C). Previous reports indicated that seed region of conserved mature miRNAs is located at position between 2–8 bp. The results at first position demonstrated a negative bias against G nucleotide for mature miRNAs with 21–22 nt length, while bias was in favor of U nucleotide for miRNAs with 22–24 nt (Fig. 2D & S2). Within seed region (nt position 2–8), there was no bias towards or against a certain nucleotide. At nt position 22, there was a bias towards U nucleotide, while against C nucleotide (Fig. 2D).

### Differential expression levels of exosomal miRNAs

**Figure 3:**
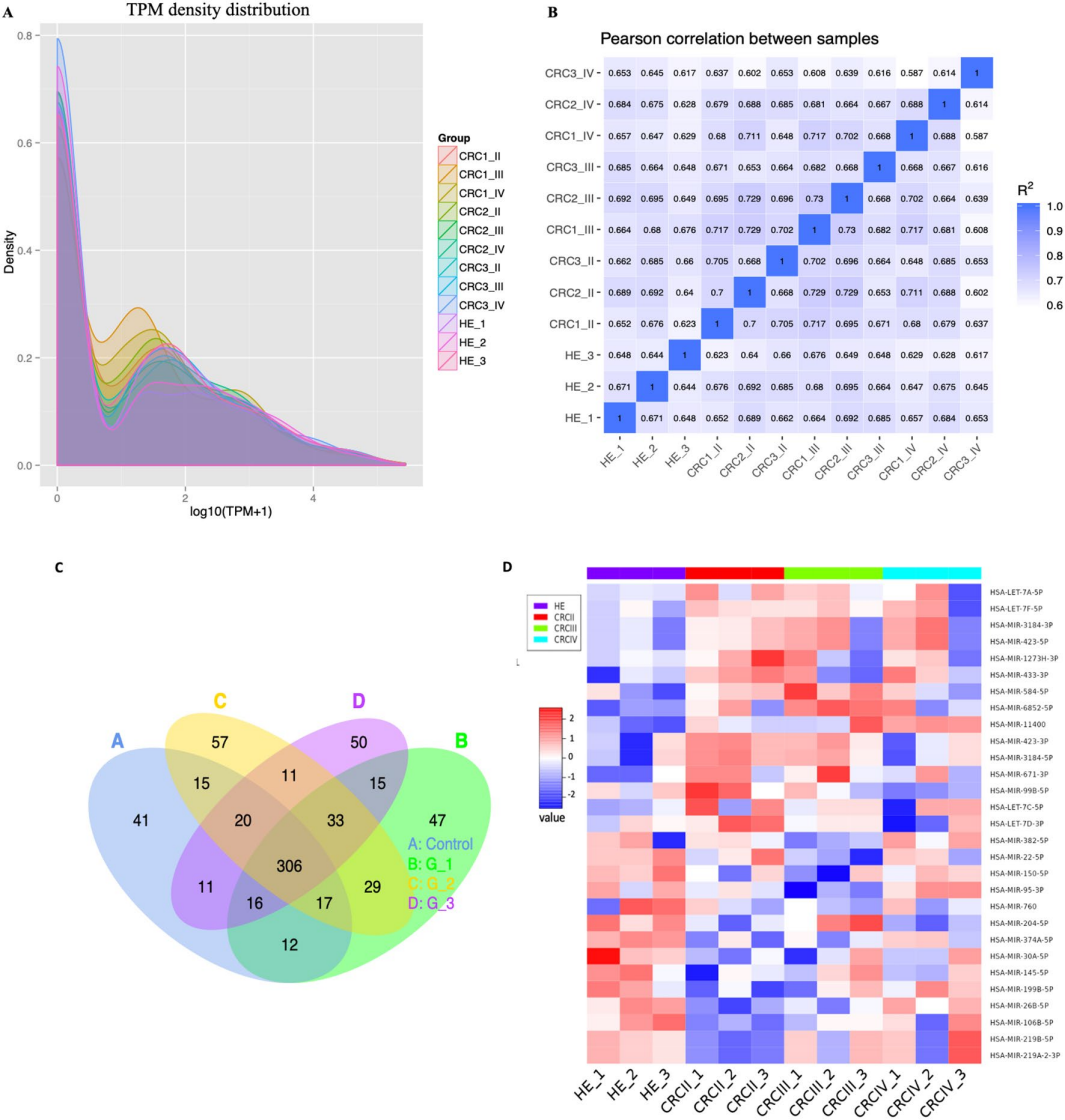
(**A**) The libraries of each group; control (HC), and CRC (II, III, and IV) are shown in different colors. The x-axis defines the log 10 (TPM + 1). The y-axis defines the probability value of the TPM distribution. TPM: transcripts per million. (**B**) Pearson's correlation coefficient of RNA-seq between each sample control and CRC (II, III, and IV). Pearson correlation coefficient square presented in shades of blue color, the ideal experiment settings is greater than 0.92, and the R2 (the square of the Pearson correlation coefficient) must be at least 0.8. (**C**) Venn diagram representing shared known miRNAs identified in each CRC group as well as healthy control. (**D**) Hierarchical clustering of 29 differentially expressed exosomal miRNAs shows colorectal cancer (CRC) patients in different stages and healthy control (HE).

Known and unique miRNAs expression in each sample are statistically analyzed and normalized by transcript per million (TPM) (Zhou et al.^25^). The formula is normalized expression = (read count*1,000,000)/lib-size (miRNA read count of sample). Quality control of RNA sequencing data was shown in TPM density distribution and Pearson correlation coefficient. The patterns of miRNAs expression of each sample were presented in miRNA TPM density distribution as shown in Fig. 3A. While the RNA-Seq correlation shown in Fig. 3B presented the biological replicate for each sample. The results showed that the correlation between same samples is similar (1), but the correlation between each sample with its group, e.g., (CRC 1, 2 and 3 of group II) were (0.7), (CRC 1, 2 and 3 of group III) and (CRC 1, 2 and 3 of group IV), was 0.6.

To identify known miRNAs in serum exosomal miRNA libraries (control and CRCII, CRCIII and CRCIV), mapped sequences were aligned to miRNA sequences of *Homo sapiens* in miRBase database. Total mapped miRNAs were annotated in each group (control and CRCII, CRCIII and CRCIV) and a total of 660 known miRNAs were detected as shown in Venn diagram of Fig. 3C (S3). Of these, a number of 306 were shared among three CRC as well as control groups. Differential expression of exosomal miRNAs between control vs. groups CRCII (G1), CRCIII (G2), or CRCIV (G3) was established using statistical criteria of fold change ≥ 2.0 and P-value of ≤ 0.05. Results indicated occurrence of 29 miRNAs with significantly different expression levels. Of which, a number of 14 miRNAs were significantly up-regulated, while 15 miRNAs were significantly down-regulated (Fig. 3D). Hierarchical clustering based on 29 DEEMs showed unclear separation between CRC groups where only group of CRC II clustered clearly and separately from a healthy control. However, each group of CRC represents a different expression pattern (Fig. 3D, S4).

### Predicted functions of differently expressed exosomal miRNAs

Target genes of differently expressed exosomal miRNAs (DEEMs) were predicted by using miRanda with present relationships between miRNAs and corresponding target genes. GO Enrichment Analysis is a technique of generating gene numbers ^26^. It begins by mapping all target gene candidates in database to Gene Ontology GO (http://www.geneontology.org/).

**Figure 4:**
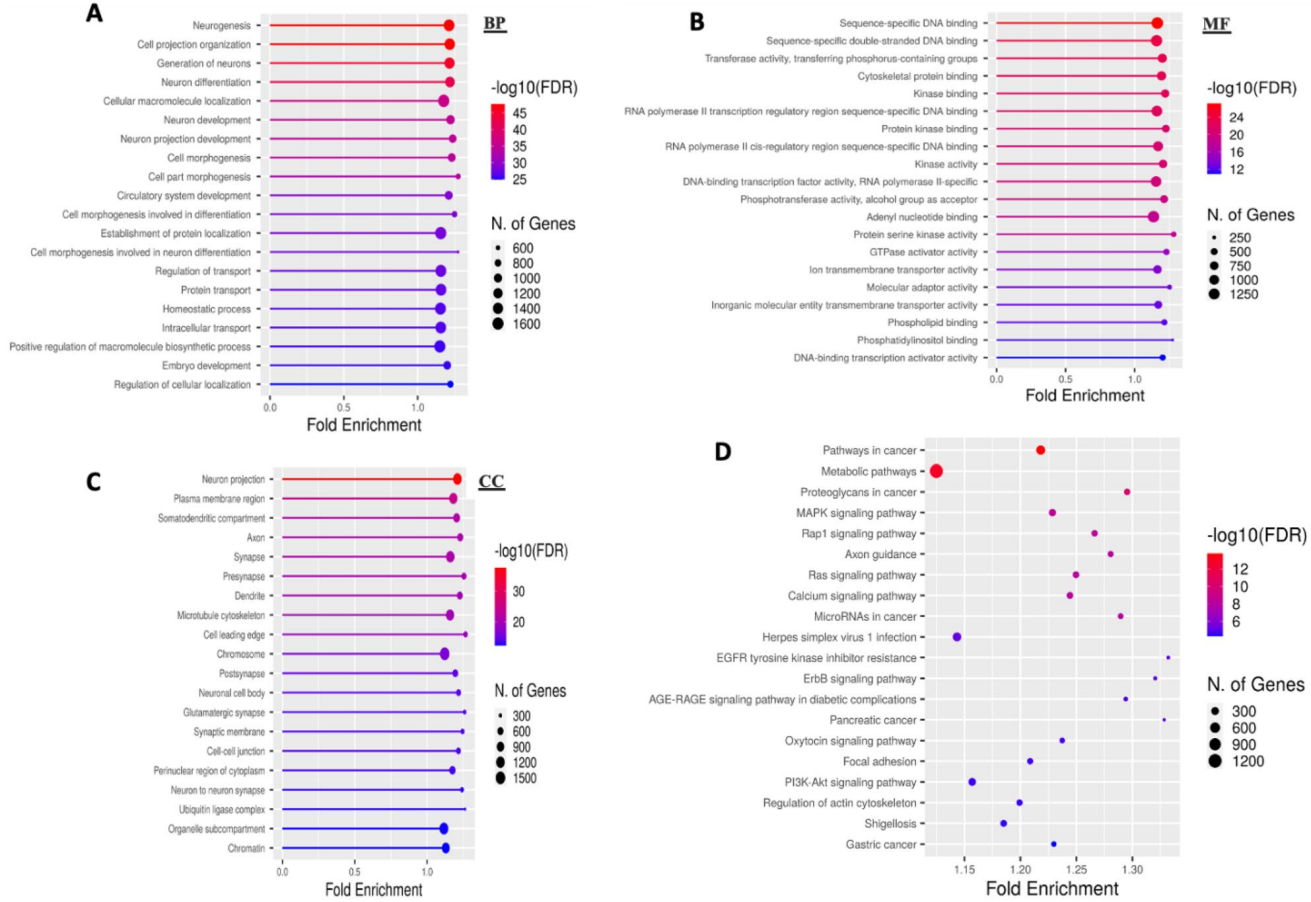
Histogram of target gene candidates. Three GO ontologies in next GO term are presented in x-axis, while number and percentage of target gene candidates annotated in this GO term are presented in y-axis (**A**–**C**). Biological process(BP), molecular function (MF) and cellular component (CC). (**D**) KEGG analysis for target genes of differently expressed miRNAs. Name of pathway is shown on y-axis, while Rich factor is shown on x-axis. Size of dots represents number of target genes, and color represents p-value.

To find significant enriched GO terms in target gene candidates, Wallenius non-central hyper-geometric distribution was used in comparison with reference gene. From 30 DEEMs, around 14,782 predicted target genes were identified by miRanda database and by applying ShinyGO v0.75 which presents all top 30 in each three classes: biological process (BP), molecular function (MF) and cellular component (CC). Probable target genes in BP class, putative target genes were highly correlated to neurogenesis, cell projection organization, generation of neurons, and neuron differentiation. While MF class was largely associated with sequencing-specific DNA binding, sequencing-specific duple-stranded DNA binding, transferase activity, and transferring phosphorus-containing groups. In CC class, probable target genes were largely connected to neuron projection, plasma membrane region, and synapse (Fig. 4A–C, S5). KEGG analysis was applied to distinguish roles of 30 DEEMs based on function of their target genes. From KEGG pathways, the top 20 high pathways were selected.

Differentially expressed miRNA target genes were enriched in metabolic pathways, pathways in cancer, proteoglycans in cancer, miRNAs in cancer, MAPK, Rap1, RAS, and several cancers were involved, ex., gastric cancer, and pancreatic cancer (Fig. 4D, S6). It will be significant in the future to functionally confirm these differentially expressed miRNA target genes.

### Identification of potential prognostic biomarkers for CRC

**Figure 5:**
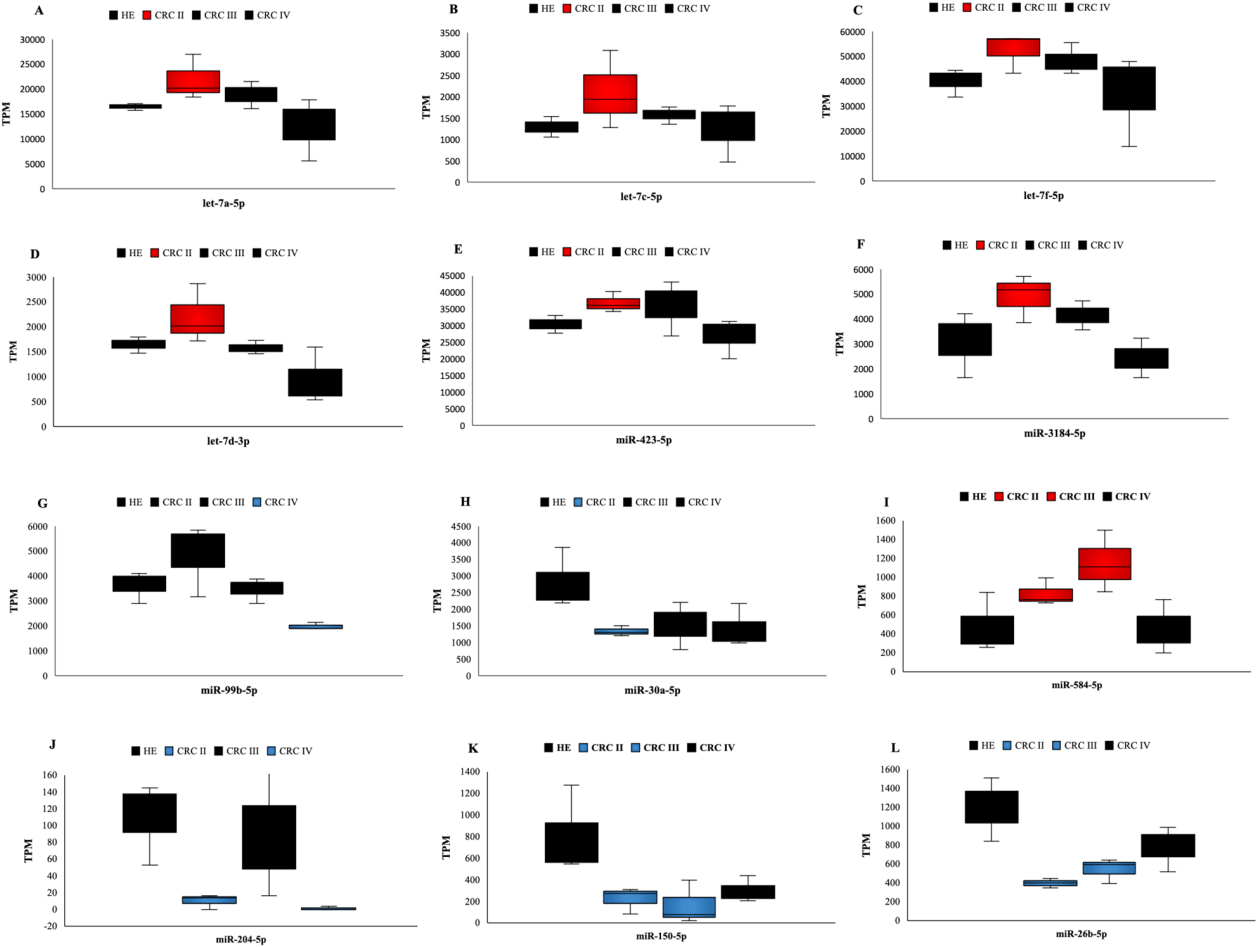
Top 12 regulated miRNAs in CRC compared with control. (**A**–**F**) miRNAs let-7a-5p, let-7c-5p, let-7f-5p, miR-423-5p, and miR-3184-5p, respectively, showed a high level of expression only in CRC stages II. The two lowest expressed in CRC. (**G**) miRNA miR-99B-5p was observed at low level of expression only in CRC stage IV compared with healthy control. (**H**) miRNAs miR-30a-5p was observed at low level of expression in CRC stages II. (**I**) miR-584-5p was up-regulated at CRC stages II and III. (**J**) miR-204-5p was down-regulated at CRC stages II and IV. (**K**, **L**) miR-150-5p and miR-26b-5p were down-regulated at CRC stages II and III. TPM; transcript per million. Red box: up-regulated miRNAs; blue box: down-regulated miRNAs; black box: miRNAs not differentially expressed.

The 12 differently expressed exosomal miRNAs (DEEMs) showed high significance (*P* value < 0.05) (**S7**). Log2 FoldChange (FC) means effective size estimated and level of miRNAs expression of CRC samples in comparison with healthy control. Positive log2 (FC) indicates up-regulation of miRNAs, while the negative log2 (FC) refers to down-regulation. The six miRNAs that were up-regulated are let-7a-5p, let-7c-5p, let-7f.-5p, let-7d-3p, miR-423-5p, and miR-3184-5p, whereas those that were down-regulated (2) are miR-30a-5p, and miR-99-5p (Fig. 5A–H). Compared with control, there was a significant up-regulation of serum exosomal miRNAs with high abundance (let-7f.-5p, miR-423-5p, let-7a-5p, miR-3184, let-7c-5p, and let-7d-3p) (Fig. 5A–F). Results of let-7a-5p, let-7c-5p, let-7f.-5p, let-7d-3p, miR-423-5p, and miR-3184 were observed at a high level of miRNAs expression (P < 0.05) in CRC II, while these miRNAs were gradually down-regulated in CRC III and IV compared with healthy control (Fig. 5A–F).

Nevertheless, exosomal miR-99b-5p and miR-30a-5p were observed at a low expression level with high abundance compared with other down-regulated miRNAs. miR-99b-5p was down-regulated only in CRC IV, while miR-30a-5p was only identified in CRC II compared with healthy controls (Fig. 5G, H). Several miRNAs were shared among CRC stages II, III, and IV including miR-150-5p, miR-204-5p, miR-26b-5p, and miR-584-5p (Fig. 5I–L).

Expression level of miR-584-5p increased progressively in CRC II and III (827 to 1153) and decreased in CRC IV to (458 TPM) close to expression level of healthy control (476 TPM) (Fig. 5I). In contrast, expression level of miR-150-5p decreased gradually from healthy control until stage III (799, 221 and 164 TPM, respectively), then increased slightly at stage IV (296 TPM), which indicates that expression level of miR-150-5p was down-regulated from CRC II to III (Fig. 5K). While miR-26b-5p showed a sharp decrease in expression level of healthy control compared with CRC patients at stage II (1191–396 TPM), then gradually increased at stages III and IV (542 and 781 TPM, respectively). That indicates that miR-26b-5p was down-regulated in CRC II and III, and expression level between these stages was steady (Fig. 5L). On the other hand, expression level of miR-204-5p was different among stages, presenting a sharp decrease from healthy control to stage II (109 and 10 TPM, respectively) with negative log2 (FC) that is three times lower in stages II than in healthy control. Interestingly, in stage III, level of miR-204-5p expression was increased slightly then decreased sharply at stage IV (88 to 1 TPM) with also negative log2 (FC) that is four times lower than healthy control (Fig. 5J).

### Gene target analysis of selected exosomal miRNAs

**Figure 6:**
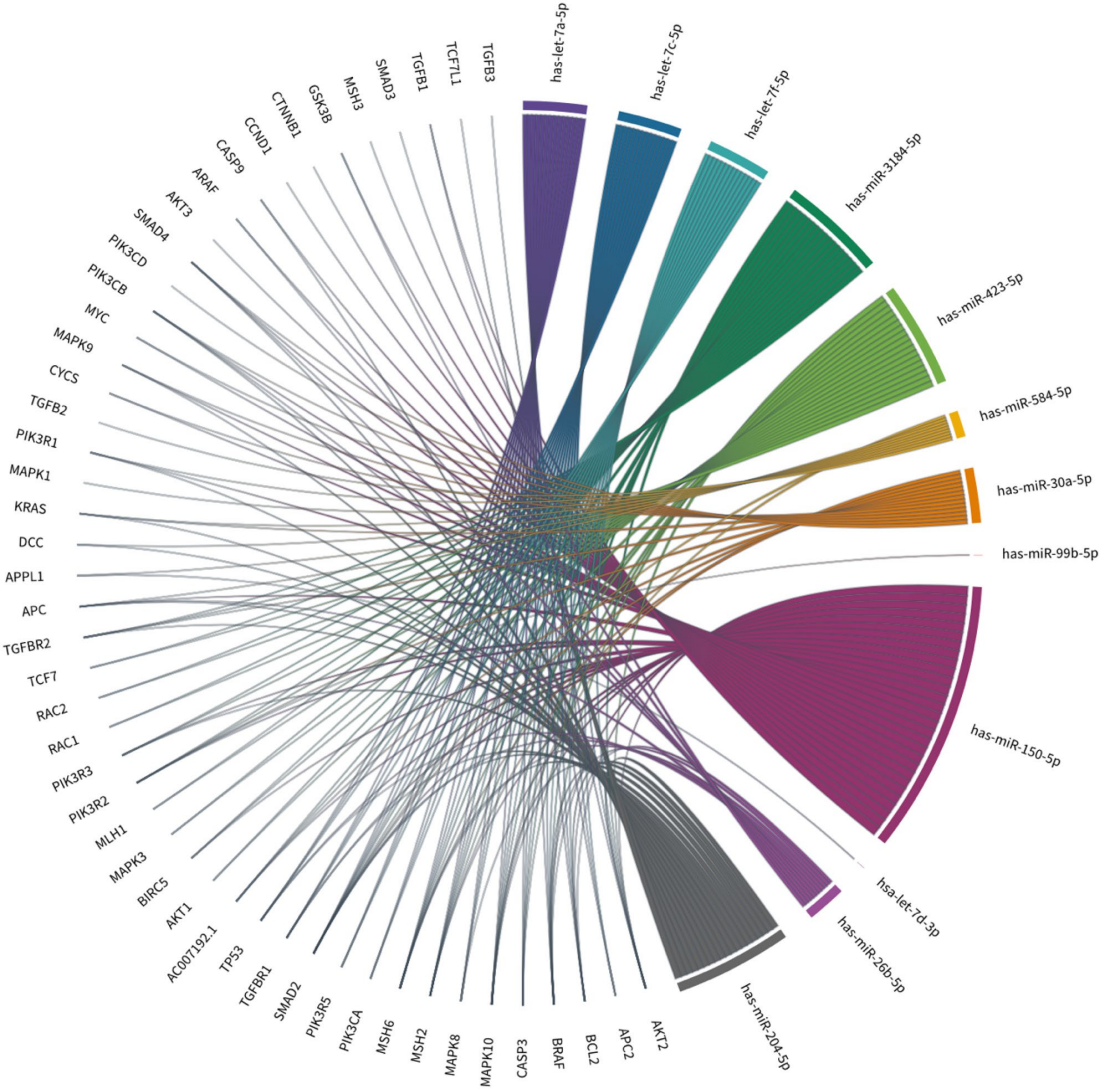
Chord-diagram of 12 differentially expressed exosomal miRNAs targeting several genes expressed in CRC. Around 49 target genes interacted with the 12 differently expressed exosomal miRNAs. miRNAs are defined in a different color, while each gene target is defined in gray color.

miRanda analysis was used to predict gene-targeted of 12 up/down-regulated miRNAs. To determine which of the 12 miRNAs could be related to CRC progression, target genes that were identified in miRanda were screened in colorectal cancer pathways, highlighting 50 genes previously associated with CRC. Results of Chord-diagram showed that all 12 exosomal miRNAs interacted with at least one gene in CRC (Fig. 6). miR-150-5p was detected to target a high number of CRC genes, around 29 out of 49 genes. While miR-9-5p, miR-30a-5p, and miR-584-5p were shown to interact with a total of 16, 13, 11, and 9 target genes. However, let-7a-5p, let-7c-5p, and let-7f.-5p were interacted with the same 14 CRC genes, including AKT2, APC2, BCL2, BRAF, CASP3, MAPK10, MAPK8, MSH2, MSH6, PIK3CA, PIK3R5, SMAD2, TGFBR1, and TP53. While let-7d-3p interacted with one gene, APPL1. In addition, miR-3184-5p and miR-423-5p were interacting with same 18 CRC genes including AC007192.1, AKT1, AKT2, BIRC5, BRAF, CASP3, MAPK10, MAPK3, MLH1, MSH2, MSH6, PIK3R2, PIK3R3, RAC1, RAC2, SMAD2, TCF7, TGFBR2. However, miR-99b-5p target only one gene, PIK3R2.

Some of the known genes that altered in CRC were highly targeted by most of the 12 exosomal miRNAs. For example, SMAD2/3 and SMAD4 were highlighted and disrupted in sporadic CRC^27^ and our result identified 11 exosomal miRNAs were interacted with SMAD2, one exosomal miRNAs with SMAD3 and four exosomal miRNAs with SMAD4. Moreover, TGFβ signaling pathways including TGFβ1, TGFβ2, TGFβ3, TGFβR1, TGFβR2 were implicated among 10 exosomal miRNAs except let-7d-3p and miR-99b-5p. Furthermore, APC and TP53 are tumor suppressor genes that are inactivated in CRC^28,29^ and were identified to interact with seven and five exosomal miRNAs respectively. Overall, these miRNAs had the largest number of interactions with commonly changed genes in CRC, indicating that they may play a role in CRC development or progression through the regulation of key genes.

### Pathway analysis for selected miRNAs

**Figure 7:**
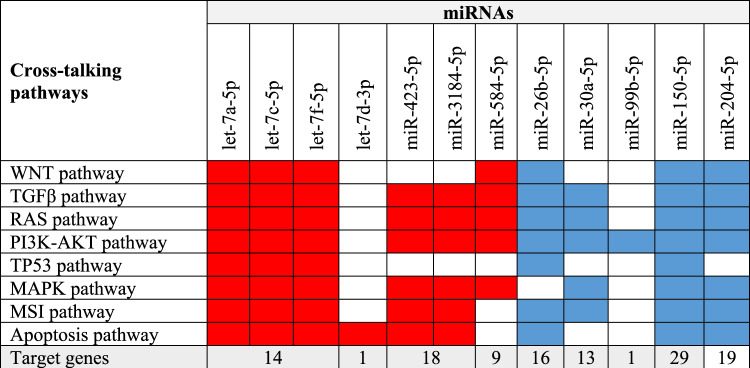
Cross-talking KEGG pathways with Colorectal Cancer pathway showing association between frequently changed pathways in CRC and the nine differently expressed miRNAs. Through KEGG database, enrichment analysis was performed on the nine miRNA-target genes. The genes interfering in each signaling pathway interacting with the miRNA-target genes were found to be associated with CRC. Number of target genes for each miRNA presented in CRC pathways is shown. miRNAs' connection with each KEGG pathway is presented in red for up-regulation and blue for down-regulation.

Pathways of 49 genes that were targeted by miRNAs were examined to determine involvement of the 12 DEEMs in CRC formation. Using KEGG database, enrichment pathway analysis indicated involvement of more than 100 pathways (*P* value > 0.05, Supplementary S3). The results showed that miRNAs play a critical role in KEGG pathways. The top highly DEEMs were identified as targeting genes related to "microRNAs in cancer” with *P* value = 4.87E^−23^ followed by “Pathways in Cancer" with a *P* value = 9.30E^−19^, MAPK signaling pathway with *P* value = 1.24E^−14^. RAS signaling pathway stood at position nine with *P* value = 3.06E^−11^, and PI3K-Akt signaling pathway stood at position 13 with *P* value = 1.59E^−10^. Also, several cancer pathways were involved in the top 30 pathways including breast cancer with *P* value = 5.70E^−10^, glioma with *P* value = 6.47E^−10^, gastric cancer with *P* value = 3.71E^−08^ and pancreatic cancer with *P* value = 6.84E^−08^. Nevertheless, colorectal cancer pathway represents a significant (*p*-value = 1.28E^−06^) but not included in the top 20 enriched KEGG pathways. Furthermore, additional signaling pathways were identified as crosstalk with CRC pathways such as WNT signaling pathway (*P* value = 7.39E^−08^), and TGFβ signaling pathway (*P* value = 9.96E^−06^). This is emphasizing that the 12 DEEMs play a significant role in CRC. These findings provide a strong connection between the 12 DEEMs and various changes that are frequently observed in CRC. Next step was to investigate where each miRNA interferes in often-changed pathways in CRC (e.g., WNT, TGFβ, RAS, PI3K-AKT, TP53, MAPK, and MSI signaling pathways)^28,30–34^. miRNA-target genes that are known to be associated with CRC were combined with genes engaged in each signaling pathway. According to these findings, majority of miRNAs were shown to be involved in at least one of these pathways. Intriguingly, various miRNAs appear to interfere in several pathways, and all 12 miRNAs appeared to target PI3K-AKT pathway. Moreover, four miRNAs (e.g., let-7a-5p, let-7c-5p, let-7f.-5p, and miR-150-5p) were shown to interact with the eight mentioned pathways. Interestingly, let-7d-3p was shown to interact with apoptosis pathway, only. Moreover, miR-204-5p presented targets in all of these pathways except for TP53 pathway. Furthermore, all up-regulated miRNAs were shown to communicate with MAPK pathway. However, TP53 pathway has lower interaction with these miRNAs. Interestingly, one miRNA (e.g., miR-99b-5p) targets PI3K-AKT pathway, only. As a result, it is possible that the changed expression of these miRNAs may participate in altering pathways in CRC through targeting several genes of these pathways (Fig. 7).

### Validation of miRNA Expression via RT-qPCR

**Figure 8:**
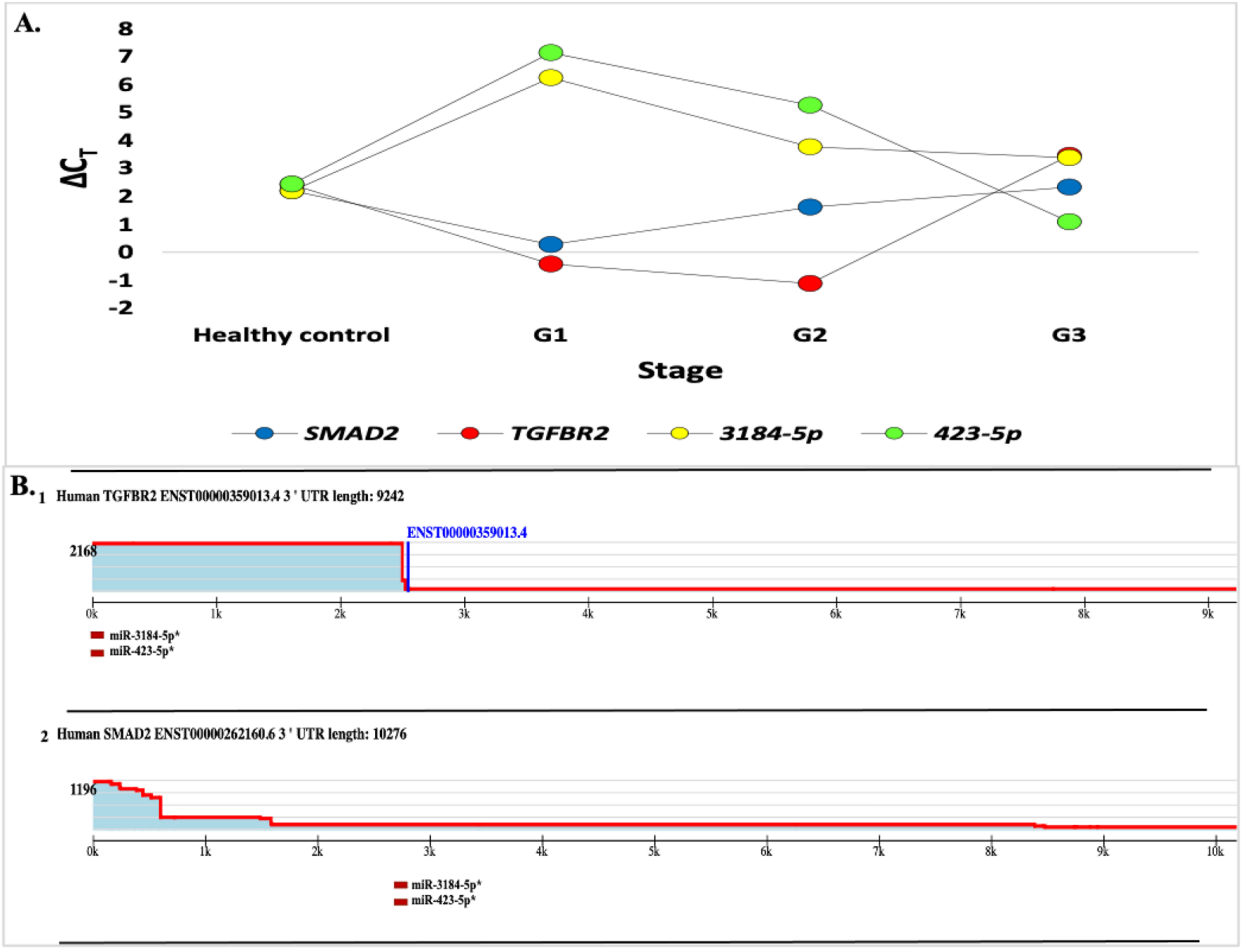
(**A**) Verification results of upregulated miRNA. The Fold Change bar graph shows expression of *SMAD2, TGFBR2*, 3184-5p, and 423-5p *genes*. (**B**) TargetScanHuman figure for the 3′ UTR of the TGFβ R2, and SMAD 2. The 3′-UTR profile on the top presents the relative expression of 3′-UTR isoforms and the total number of 3P-seq reads. The profile presents Gencode annotation (red line), the conserved sites of the predicted target of miRNAs (boxes). The predicted site of miRNA up-regulation is miR-423-5p and miR-3184-5p(red boxes).

We analyzed RT-qPCR data derived from a study conducted to validate differentially expressed miRNAs in patients vs. healthy controls of the deep-sequencing data, we performed qRT-PCR on the identified. The validation was conducted for two miRNAs namely miR-3184-5p and miR-423-5p using the same RNA samples of the CRC patients except for one patient from stage IV (the result was undetermined). According to the present findings, these two miRNA were upregulated and significantly increased in both stage II (G1) and stage III (G2) when compared to the healthy control. In contrast, their target genes SMAD2 and TGFER2 were downregulated in stage II (G1) and stage III (G2) compared to healthy control. However, the expression of these genes gradually increased, and inversely with miR-3184-5p and miR-423-5p, which were decreased in stage VI (G3) (Fig. 8A).

It was previously reported that TGFβ signaling pathways are involved in multiple aspects of CRC progression; we hypothesized that the low expression of several genes in this pathway, such as TGFβRII, and SMAD2 in CRC, could be due to the up-regulation of miRNAs that target this pathway. The map of each gene in the TGFβ signaling pathway and a list of expressed miRNAs expected to target these genes based on TargetScan prediction are displayed in (Fig. 8B 1&2). The TargetScan database^35^ presented the TGFβRII and SMAD3 genes were targeted by both miR-423-5p and miR-3184-5p with one poorly conserved site.

## Discussion

CRC initiation and progression are intimately linked to genetics and several environmental variables^36^. Because patients with advanced colorectal cancer (CRC) typically have a bad prognosis, detecting CRC early becomes is extremely important^36,37^. Development of useful biomarkers is critical to improve accuracy of CRC diagnosis and treatment. Researchers have discovered that miRNA plays a crucial role in the pathogenesis and progression of CRC^38^. Use of miRNAs properties that are recognized at early stages in blood circulation system, or early cancer detection, yielded highly encouraging results. However, based on differences in miRNA expression amongst populations, environments, nutrition, etc., findings of discovered miRNA expression may change^39^. Exosomal miRNA expression profiles have been reported in several studies to control various target genes that affect recipient cell function. Thus, exosomal miRNAs with specific properties are prospective biomarkers that can be employed as a diagnostic and prognostic tool for CRC or could be used as potential targets for therapeutic intervention of CRC metastasis.

In the present work, the importance of serum exosomal miRNAs in CRC patients compared with a healthy control group was examined using miRNA sequencing datasets. Detecting miRNAs in serum samples was discussed in this study to effectively discriminate malignancy from healthy controls and delivering panels of miRNAs as predictive biomarkers for improved patient classification, specifically for CRC stages II, III and IV. Towards this, deep sequencing was utilized to identify miRNA expression levels in serum samples at different stages of CRC. This method provides potential benefits in universal miRNA expression research, but it also introduces additional data analysis issues, since quantity of data obtained after deep sequencing involves millions of reads that must be mapped to genome and normalized^36^. The majority of miRNA expression alterations in CRC appears to begin during normal mucosa-to-cancer development and are sustained throughout metastatic transition^40^, which is consistent with our findings.

The sequencing data and bioinformatics analysis in our study provided a strong finding of mature exosomal miRNAs. A number of 660 mature miRNAs were found at different stages of CRC for patients that were successfully diagnosed. From the 29 differentially expressed miRNAs found in CRC patients compared with healthy controls, 12 regulated miRNAs were selected depending on their high abundance levels. The highly up-regulated miRNAs (e.g., let-7a-5p, let-7c-5p, let-7f.-5p, let-7d-3p, miR-423-5p, miR-3184-5p, and miR-584-5p) and down-regulated miRNAs (e.g., miR-30a-5p, miR-99-5p, miR-150-5p, miR-26-5p, and miR-204-5p) were analyzed further (Fig. 3A–D). We predicted that the 12 changed miRNAs may have influenced several critical target genes in CRC that lead to tumor occurrence and growth. Most of diagnostic exosomal miRNAs (e.g., let-7a-5p, let-7c-5p, let-7f.-5p, let-7d-3p, miR-423-5p, miR-584-5p, miR-30a-5p, miR-99-5p, miR-150-5p, miR-26-5p and miR-204-5p) have been discovered to be circulating in blood serum.

In addition, it is hypothesized that exosomes have a particular way for releasing nucleic acids into extracellular fluid and include miRNA species that have been carefully packed to migrate to other cells for knocking out cognate target genes^41–43^. Accordingly, gene target prediction software was used to suggest probable target genes based on sequence pairing. Predicted target genes of these highly expressed exosomal miRNAs strongly relate to cancer pathways that involve CRC pathway. The target genes in CRC pathway include tumor suppressor genes, oncogenes, DNA repair genes, and multiple critical signaling pathways such as TGFβ that are directly linked to CRC function in which this study focuses. KEGG analysis showed that differentially expressed miRNAs are included in cancer signaling pathways and gene ontology (GO) analysis specified the top expressed miRNAs involved in molecular functions, cellular components, and biological processes categories.

Transforming growth factor β (TGFβ) signaling pathway has been identified to promote growth inhibitory of tumorigenesis. However, dysregulation of TGFβ signaling have been reported in several studies to contribute to cancer proliferation, metastasis, and angiogenesis (Feng and Derynck^21^; Massagué et al*.*^20^). miRNAs have been shown to influence formation and activation of TGFβ signaling pathway, and miRNAs/TGF interaction was also shown to play a significant role in development of several malignancies, including lung, breast, pancreatic, gastric and CRC (Soleimani et al*.*^22,23^). Oncogenic miRNAs have been found in several cancer types to enhance proliferation, invasion, or resistance to chemotherapeutic treatments by targeting TGFβ receptors, particularly TGFβRII (Suzuki^44^).

By focusing on target gene sequences, let-7 family (let-7a-5p, let-7c-5p, and let-7f.-5p) has a highly similar sequence and shares the same seeds region (3’GAUGGAG’5), leading to targeting identical genes. Members of let-7 family (let-7a-5p, let-7c-5p and let-7f.-5p) share identical seed sequence, which represents highly conserved region that is used to recognize target mRNAs sequences^45^. This indicates that function and targets of let-7 family may be either similar or different^46^. Results of the present work showed that let-7 family members (let-7a, let-7c, let-7f) shared same target genes in CRC pathways. Let-7 family is considered as an essential tumor suppressor miRNA that regulates several oncogenes^46^. The study presented that through low level of let-7, several signaling pathways are enriched such as cell cycle and cellular proliferation, which promote cancer progression^47^. Down-regulation of let-7 negatively controls MYC and RAS expressions that are recognized to be important regulators for CRC progression^47,48^. Through direct binding, let-7 is able to inhibit RAS expression and hence control MAPK and PI3K/AKT pathways^49^. Kawata and colleagues determined that serum exosomal levels of let-7a were higher in primary CRC patients than in healthy controls, while much lower following tumor resection. In addition, authors demonstrated that colon cancer cell lines produced these miRNAs at considerably greater levels than normal colon cell lines^50^. Furthermore, a low level of let-7c expression in primary cancer tissues of CRC relates to metastases, progressed TNM stages, and poor prognosis of patients. Let-7c acts as a tumor suppressor for CRC development and metastasis by disrupting mRNAs of MMP11 and PBX3^51^. While let-7f has been confirmed to be down-regulated in both plasma and stool samples of patients with early stages of CRC (I and II) compared with healthy control^52^. Co-expression of both let-7d-3p and let-7d-5p was found to contribute to colon cancer by exclusively inhibiting KRAS by let-7d-3p, and promoting IGF1R and THBS1 expression by let-7d-5p^53^. The study by Ohshima and colleagues demonstrated that cancer cell lines that produce exosomes load a high amount of let-7 family. It is proposed that these processes occur to release tumor suppressor miRNAs and support ongoing tumorigenesis and metastasis^54,55^. Referring to our results, where let-7 is a tumor suppressor miRNA, its abundance in serum primary CRC stage seems to be a predictor of enhanced carcinogenesis and metastatic action in malignant tissue, offering a novel perspective as to how these biomarkers are regulated (Fig. 5A–D).

**Figure 9:**
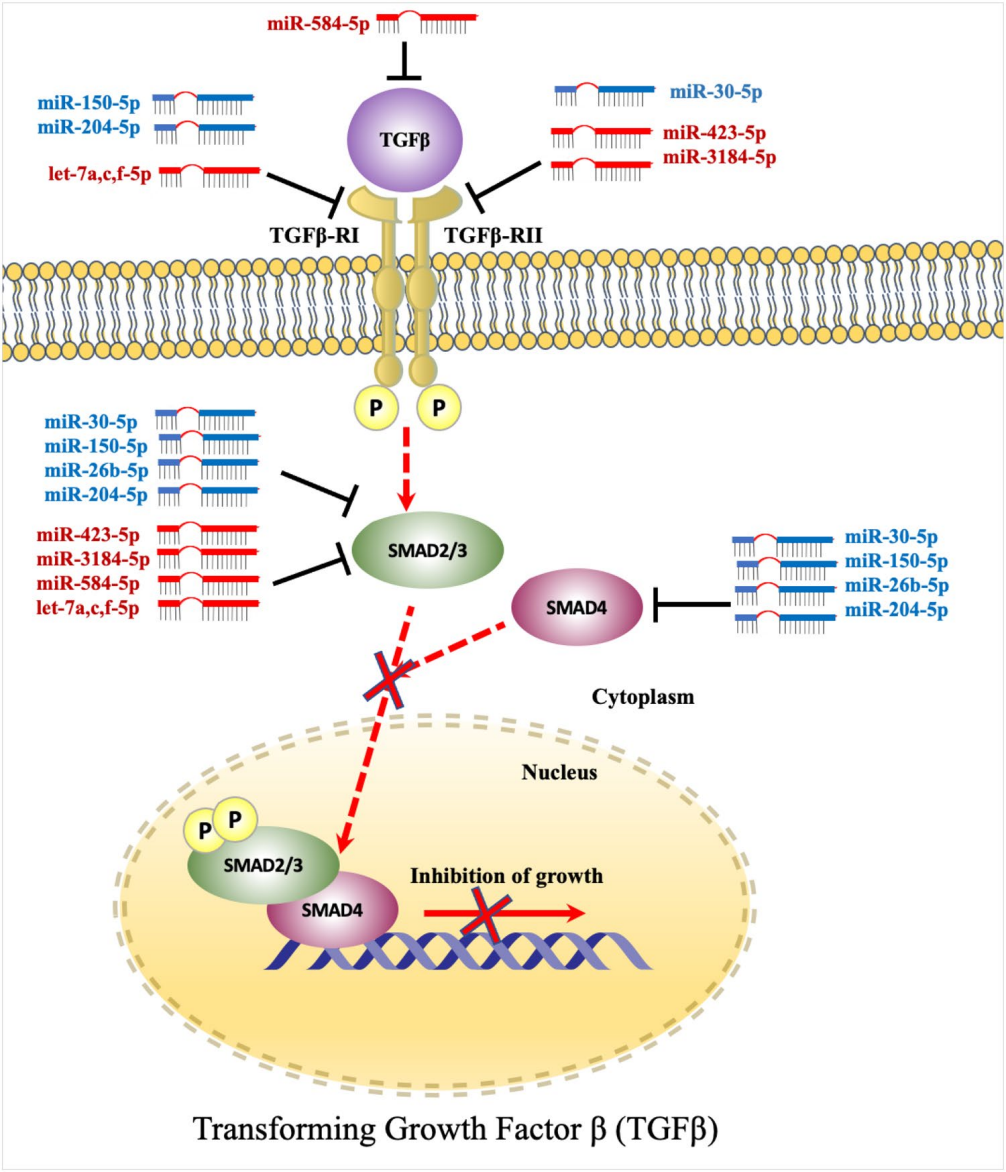
TGFβ signaling pathway. TGFβRI, and TGFβRII are phosphorylate SMAD2 and SMAD3 after binding to TGFβ ligands. Activated SMAD2 and SMAD3 subsequently form a complex with SMAD4. SMAD complex is a nucleoprotein that controls cell proliferation, apoptosis, metastasis, and angiogenesis, as well as modulating target gene expression and tumor formation. MiRNAs target TGFβ, TGFβRI, TGFβRII, SMAD2/3 and SMAD4 leading to disrupt inhibition of growth in TGFβ signaling pathway. Overexpressed miRNAs (red color) and downexpressed miRNAs (blue color). Direct regulation is indicated by red dashed arrows and lines.

By bioinformatics analysis, we identified that TGFβ signaling pathways including TGFβRI and SMAD2 are targeted by let-7 family (Fig. 9). However, another study demonstrated that overexpression of let-7a interacts with TGF-β1 and SMAD4 and affects cervical cancer cells^56^. In addition, TGFβRI was regulated by let-7, which could play a significant role in TGFβ signaling activity at tumorigenesis stages^57^. However, no other gene targets for these miRNAs were detected. The results of miR-423-5p and miR-3184-5p indicate that they are significantly up-regulated in serum exosomal miRNAs at CRC II compared with control. However, these two miRNAs were down-regulated at CRC stage III and IV. miR-423-5p targets CRC pathways, particularly, tumor suppressor genes *SMAD2/3* and *TGFβR2*. It has been identified that miR-423-5p and miR-3184-5p share same target genes because they have the same seed sequence (3’CGGGGAG’5)^58^. Morover, miR-423-5p and miR-3184-5p were significantly reported by several researches as oncogene and are involved in carcinogenesis, tumor development, therapeutic sensitivity, and prognosis in different types of cancer^59–63^. In glioblastomas, miR-423-5p acts as tumor promoter, leading to proliferation, invasion, and angiogenesis of cancer^60^. Plasma level of miR-423-5p has previously been identified as a possible biomarker for CRC occurrence^60^. While miR-3184-5p has been linked to formation of malignancies in a variety of human cancers. High expression level of miR-3184-5p was identified in hepatocellular carcinoma (HC) cell lines, according to miRNA array data^64^. Furthermore, exosomal miRNA-3184-5p was shown to be able to distinguish non-small-cell lung cancer (NSCLC) at early stages from normal controls^65^. miR-3184-5p was also shown to be highly elevated in breast cancer cells and operates as an oncogene in obesity-related breast cancer due to its influence on cell proliferation and invasion^61^. By predicting target genes for these miRNAs, it was demonstrated that certain target genes play a significant role in both activation and inhibition of CRC. Furthermore, up-regulation of miRNAs acts as oncogenic miRNA targeting certain tumor suppressor genes related to CRC initiation and development^22^. More specifically, *TGFβR2*, *SMAD2*, and *SMAD3* genes are key components of TGFβ that regulate growth inhibitory, and abnormality of these genes contributes to tumorigenesis^44^. Inactivation of these genes results in dysregulation of TGFβ signaling, which leads to loss of growth inhibitory effect^21^. Our results suggest that miR-3184-5p and miR-423-5p may accumulate in these genes and disrupt transcriptional process of TGFβ signaling pathway at this stage (Fig. 5E, F). Also, up-regulation of exosomal miR-423-5p targeting GREM2 among TGFβ pathway enhances therapeutic resistance as well as contributes to malignant progression of prostate cancer (PC)^66^.

Moreover, miR-99b-5p and miR-30a were down-regulated in CRC in the present work and contributed to different cancer progression stages^67–72^. miR-99b-5p was down-regulated in CRC stage IV comparing to healthy control and was identified in our data to only target *PIK3R2* gene in CRC pathway. The study by Li and colleagues showed that miR-99b-5p was overexpressed in primary tumors of patients with CRC and that suppressing this miRNA promotes cell migration and overexpression of mTOR of CRC cell lines. Also, authors suggested that miR-99b-5p is collaborated with more prolonged overall survival of patients and acts as a tumor suppressor in metastatic CRC by targeting mTOR. This indicates that miR-99b-5p may interact with mTOR and function as a predictive factor as well as a therapeutic target for anti-metastatic CRC patients^72^. Moreover, miR-99b-5p is associated with numerous malignancies, including colon cancer^69^. In gastric cancer, miR-99b-5p suppressed proliferation via negatively modulating insulin-like growth factor 1 receptor (IGF-1R) and stimulating AKT pathway^73^. It was identified that exosomal miR-99b-5p has been increased after tumor surgery, representing that exosomal miR-99b-5p expression level negatively influences tumor survival. As a result, miR-99b-5p (Fig. 5G) is identified as a possible clinical diagnostic biomarker capable of reliably and promptly distinguishing CRC patient^69^. It was identified that expression of TGF-β is affected by specific miR-99 family members such as miR-99a-5p, but not miR-99b-5p, and target *SMAD2* and *SMAD4* genes in acute megakaryoblastic leukemia (AMKL)^67,74^.

According to previous research, miR-30a expression is related to a variety of cancer types, such as thyroid, breast, and gastric cancers, as well as CRC^68,70,75,76^. miR-30a acts as a tumor suppressor in majority of these malignancies by modulating conserved target genes. miR-30a targets insulin receptor substrate (IRS2), phosphoinositide 3-kinase catalytic subunit delta (PIK3CD), and integrin β3 in CRC to inhibit proliferation, migration, and invasion^24,71,77^. Interestingly, low expression of miR-30a was in all stages but in stage II was more significant which indicates that this miRNA has a significant impact in this stage rather than other stages. It was demonstrated that miR-30a overexpression decreased migration and invasion and controlled E-cadherin and vascular endothelial growth factor (VEGF) expression by suppressing *TM4SF1* gene. The findings indicate that miR-30a is related to progression stage and lymph node metastasis of CRC and that it might be a new promising source for diagnosing and therapy of CRC. Interestingly, our study identified that down-expression of miR-30a was represented in CRC stages III than in stages III and IV (Fig. 5H), which demonstrates that miR-30a plays a critical role in early stages of CRC development than in the later stages^70^. These findings suggest that miR-30a is negatively related to carcinogenesis and lymph node metastasis and may function as a tumor suppressor in CRC. Additionally, in CRC patients with severe clinical characteristics, low serum miR-30a-5p expression was more common. Moreover, CRC patients with high serum miR-30a-5p expression had substantially longer overall survival rate than those with low expression. Finally, analyses demonstrated that serum miR-30a-5p expression was a significant predictor of survival in CRC patients^68^.

Moreover, miR-30a-5p targets TGFβ signaling pathway, which could be disrupted by multiple processes related to tumor invasion. As described in anaplastic thyroid cancer (ATC), down-regulated miR-30a-5p correlates with inhibition of TGFβ signaling. This miRNA was associated with induced mesenchymal-epithelial transition (MET) and ATC cells invasion^78^. Expression of *SMAD2* and *TGFBR1* genes, which were elevated in most primary anaplastic thyroid cancer (ATC), was regulated by miR-30 in ATC-derived cells confirming involvement of TGF signaling in modifying MET/epithelial–mesenchymal transition (EMT)^79^. Based on these findings, miR-30a-5p is proposed to have a potential role as a novel biomarker for CRC diagnosis and/or prognosis. On the other hand, TGFβ has been reported as a tumor suppressor in the early stages but a tumor promoter in the later stages^80^. Some overexpressed miRNAs suppress anti-proliferative signals by targeting this pathway. In contrast, the TGFβ pathway, in collaboration with BRAF, causes EMT, migration, and invasion in later stages^80^. Our results presented that TGFBR2 has also interfered with miR-30a-5p, which could be a novel biomarker that can be studied and investigated further.

Some functions of these highly expressed miRNAs have been studied, where miR-584-5p targets some tumor suppressor genes including *STAT1*, *PTEN*, and *cyclin D1*^81^. The miR-584 was found in the SW620 colorectal cancer cell line to interfere with binding of hnRNP A1 and CDK6. The study suggested that overexpression of miR-584 acts as a tumor suppressor, which can disrupt binding between hnRNP A1 and CDK6 mRNA, leading to apoptosis. This connection might be replicated for different miRs, which could lead to creation of new cancer treatment techniques^82^. Also, miR-584 was shown to be up-regulated in CRC and greater levels of this miRNA were only previously seen in colon adenoma, not carcinoma^83^. Another research revealed that miR-584 was overexpressed in the early stages of gastric cancer (GC) and then down-regulated in later stages. Their findings showed that high levels of miR-584 expression may be essential for primary tumor formation and gastric carcinogenesis, then, this miRNA undergoes low levels for tumor progression and invasiveness in the metastasis stage^81^. Furthermore, the latter study suggested that miR-584 has the potential to be a diagnostic biomarker for early identification of GC as well as a predictive factor for predicting patient clinical outcomes. Another study indicated that in the presence of FF/CAP18 treatment, which inhibits proliferation of colon cancer cell lines, exosomes release miR-584-5p at a high level^84^. Moreover, the latter study suggested that miR-584-5p may act as a tumor suppressor and can be a potential target to inhibit tumor progression. The study of Konishi and his team suggested that overexpression of miR-584-5p suppress the interaction between heterogeneous ribonucleoprotein A1 (hnRNP A1) and CDK6 mRNA, which induce apoptosis of CRC cell line in malignant transformation stage^82^. All the latter findings support our prediction that miR-584-5p is up-regulated in CRC stage (II and III), then, down-regulated at stage IV compared with healthy control, which indicated that miR-584-5p is highly expressed during carcinogenesis and, then, decreased to a lower level of expression during invasion and metastasis (Fig. 5I). Moreover, miR-584 targets *SMAD2*, and *TGFβR2* genes leading to loss of growth inhibitory and induction of proliferation and angiogenesis of several malignancies, including CRC^20,21,23^.

MiR-150-5p was proven to be interfering in several cancers, including thyroid cancer cells (TCC), lung squamous cell carcinoma cells (LSCC), prostate cancer cells (PCC), lung, breast, pancreatic and esophageal cancer and CRC (Fig. 6)^69,85–91^. The miR-150-5p targets *MAP3K12* gene that inhibits the proliferation and invasion of PCC and LSCC^85,88^. Moreover, following surgery, the expression of exosomal miR-150-5p was elevated, showing that tumor retention may have a detrimental impact on miR-150-5p expression^69^. Exosomal miR-150-5p has been reported to inhibit the proliferation of CRC cells, assisting as a potential novel biomarker for CRC diagnosis^69^. Another study found that a low expression level of miR-150 in colorectal tissue was associated with poor survival and responsiveness to chemotherapy, demonstrating that miR-150 expression levels were correlated to CRC. It is proposed that miR-150 be examined as a possible biomarker for CRC prognosis and treatment outcome^90^. On the other hand, in breast cancer cells, increased miR-150 expression has been shown to boost clonogenicity and cell proliferation, while reducing apoptosis^92^. Various studies have also shown that miR-150 functions as a tumor suppressor and a promising prospective biomarker by altering expression of several genes associated with CRC progression, including *c-Myb*, *c-FLIP*, and *VEGFA*^90,93,94^. A previous study was done to clarify the connection between miR-150 and β-catenin in CRC. It revealed that overexpression of miR-150 inhibited the viability, proliferation, and colony formation of SW480 and HT-29 cells by targeting the Wnt/β-catenin pathway^95^. The in vivo study presented that miR-150 inhibits CRC development through regulating β-catenin. Levels of β-catenin, c-MYC, and CCND1 were negatively correlated with miR-150 overexpression suggesting that miR-150 might decrease CRC growth and operate as a tumor suppressor by blocking β-catenin pathway^95^. Interestingly, a study of the TGFβ/SMAD signaling pathway in hepatic stellate cells (HSCs) revealed that inhibiting SMAD4 by the Smad4shRNA vector enormously improved miR-150 expression levels^96^. Overall, these findings support our result that found that down-regulation of miR-150 affects other downstream signaling pathways, especially TGFβ pathways (Fig. 5K). However, this miRNA was significantly down-regulated in stages II and III, not IV, suggesting that the down-regulation of miR-150 negatively regulates CRC progression and development.

Furthermore, miR-26b expression was found in various cancers, including neck squamous cell carcinoma, osteosarcoma, cell lung carcinoma, gastric cancer, ovarian cancer cell and CRC, which can be used as prognostic predictor^97–102^. The previous studies proved that miR-26b associates with CRC by interacting with different set of target genes^82,102–106^. It was recognized that miR-26b expression was elevated in serum and tissues during the progression of inflammatory bowel disease-related tumorigenesis^107^. Nevertheless, investigations have found that low level of miR-26b expression was detected in CRC tissue only, not in normal tissue, indicating that miR-26b reduction may contribute to CRC tumorigenesis^102^. Surprisingly in CRC cell lines, miR-26b correlates with increased invasion and metastasis and contributes to lymphatic metastasis. This result appears to be paradoxical although miR-26b expression is more expressed in normal cells than in malignant tissues. The study also determined that PTEN and WNT5A, as tumor suppressors, were inhibited by miR-26b, however, expression of miR-26b is a predictor of poor prognosis following carcinogenesis, which might be an indication of miRNA tissue-specificity^103^. The present study also suggests that instead of comparing the expression levels of miR-26b in CRC cells and normal colorectal mucosal epithelial cells, identifying the effect of various miR-26b expression levels on CRC cells could be more efficient. This miRNA may have a variety of roles depending on types of cells, and normal colorectal mucosal epithelial cells and CRC cells are the examples. Therefore, determining the function of miR-26b in CRC requires more than comparing the expression of miR-26b in CRC cells versus normal cells. Alternatively, tracking of miR-26b expression should simultaneously be in the same CRC cell lines^103^. Moreover, miR-26b was found to induce a stem cell-like phenotype in a fraction of CRC cells, which indicates that miR-26b acts as a regulator of CRC invasion and metastasis^103^. Interestingly, overexpression of miR-26b-5p plays a significant impact in chronic lymphocytic leukemia (CLL) development. It was determined that overexpression of miR-26b-5p disrupts the TGFβ/SMAD signaling pathway in CLL cells by targeting *SMAD4* gene, leading to a reduction expression of p21-Cip1 kinase inhibitor and increased expression of c-MYC. This study identified miR-26b-5p as a novel molecular pathway relating CLL development to TGFβ regulation and a novel treatment method for CLL^108^. We can predict that miR-26b-5p could play a significant and key role in CRC. This includes low expression for progression in stages II and III, while for metastasis and invasion in stage IV. The current study presented that overexpression of miR-26b-5p contributes to preventing chondrocyte senescence and osteoarthritis (OA) by targeting asporin mRNA. Furthermore, asporin promotes chondrocyte senescence and exacerbates cartilage degradation by blocking the TGFβ1/SMAD2 pathway, and hence plays a role in OA. The miR-26b-5p/asporin/SMAD2 axis represents a prognostic biomarker and a possible therapeutic target in OA, according to these findings^98^. Our study demonstrated that miR-26b-5p was significantly decreased in stages II and III compered to control but still this miRNA has low expression in stage IV and no data for miR-26b-5p and TGF-β. In addition, our study supported prior research that found that miR-26b has biological impact on CRC development (Fig. 5L).

In addition, miR-204 was shown in the present study to be significantly down-regulated in CRC tissues compared with surrounding normal tissues. Previous research has found that miR-204 is often down-regulated in many different malignancies, indicating that miR-204 plays a common function in human carcinogenesis^109–112^. Moreover, previous research has found that miR-204 suppresses a variety of cancerous tumors via various signaling pathways, indicating that it may have distinct impacts on tumorigenesis. In the cases of pancreatic cancer and renal clear cell carcinoma, miR-204 might inhibit tumor growth^111,113^. Also, endometrial cancer, liver cancer, glioma, colon cancer, gastric cancer, intrahepatic cholangiocarcinoma, non-small cell lung cancer (NSCLC) and head and neck malignancies are all identified to be repressed by miR-204^109–119^. Furthermore, miR-204 plays a significant key role in the sensitivity of CRC to 5-Fu chemotherapeutic drugs by targeting high mobility group protein A2 (HMGA2). In a previous study, overexpression of miR-204 was detected to suppress proliferation of SW480 cell lines and increase response sensitivity with a 5-Fu dose, indicating that miR-204 might be used as a treatment for 5-Fu-resistant CRC^120^. Another study demonstrated that low expression of miR-204 correlates to colon cancer pathogenesis, CRC progression, and drug resistance through overexpression of angiopoietin-like protein 2 (ANGPTL2). However, up-regulation of miR-204 is accompanied with down-regulation of ANGPTL2 that led to induced apoptosis and inhibited cell proliferation^121^. Furthermore, this study determined that lower level of miR-204 expression correlates with higher TNM stages and elevated ANGPTL2 expression contributing to poor prognosis in CRC^121^. According to a previous study, overexpression of miR-204 in CRC cells significantly suppressed cell growth, lowered the ability of tumor progression of CRC cells in animals, impacted the intestinal cancer cell migration and invasion, and increased the responsiveness of cells to targeted therapies^109^. Prior studies have shown that miR-204 is also linked to CRC via interfacing with a broad set of target genes such as *HMGA2*, *CXCL8*, *RAB22A*, *TPT1*, *PCAT6* and *CREB1*^120,122–124^. Other studies presented the role of miR-204-5p in interfering with TGFβ signaling pathways through regulating *Six1* and *SMAD4* genes in airway smooth muscle cells of asthma and human posterior capsule opacification, respectively^125,126^. These findings indicate that miR-204-5p expression was dramatically decreased in tumor tissues and cells of colorectal carcinoma patients in several studies, suggesting that miR-204-5p may function as a tumor suppressor in CRC. Our results, interestingly, align with these results, which proved that miR-204 -5p is valuable in malignancies, particularly CRC (Fig. 5J). Thus, it is an opportunity to study the role of miR-204-5p with TGFβ in the development or suppression of CRC.

The results also suggest that the highly expressed exosomal miRNAs, e.g., let-7a-5p, let-7c-5p, let-7f.-5p, miR-423-5p and miR-3184-5p, are connected to cancer and are potentially important in CRC by targeting TGFβ, which is the important signaling in the CRC pathway (Fig. 8). However, lower expressed exosomal miRNAs, e.g., miR-26b-5p, miR-150-5p, miR-204-5p, and miR-30a-5p, may contribute to the progression of CRC by missed targeting oncogenes. Only let-7d-3p and miR-99b-5p were not involved in this pathway. While an additional study is needed to validate the target genes and investigate the downstream implications. Exosomal miRNAs target mRNA species that are dysregulated in CRC patients, and functional pathway analysis revealed extremely significant connections with particular CRC molecular pathways. This builds confidence that miRNA biomarkers of CRC identified during our approach likely to be fully justified.

Significant correlation represents more than half of the statistically meaningful associations, indicating that miRNA and target gene expression are usually moving in opposite directions. Although an increase in miRNA levels should decrease target levels (or vice versa), it is widely known that miRNAs are dynamic gene expression regulators that inhibit protein translation rather than mRNA degradation^127–133^. The miRNAs' biological significance appears by area of binding to the conserved seed region, whether at 3p or 5p end. This indicates that miRNAs with -3p or -5p have a significant role in biological processes and can contribute to these processes in several ways and expression patterns^53,134–137^.

It is challenging to determine the relative relevance of high versus low miRNA expression since the absence or abundance of miRNAs might reflect equally relevant biological control signals. Over-expression of miRNAs might indicate that these miRNAs play vital roles as regulators of downstream targets of biological pathways. Furthermore, these miRNAs likely interfere with signaling pathways known to be aberrant in CRC, including TGFβ, TP53, KRAS, BRAF, MAPK, and BAX. Several investigations have also shown that miRNAs play a critical role in modulating these pathways^22,30,57,138–141^, which confirm our findings. Moreover, it was demonstrated that several dysregulated miRNAs in CRC are analytically mapped to targets implicated in cancer progression pathways^142^.

In conclusion, serum exosomal miRNAs profile may in the future become valuable biomarkers in the management of CRC. They represent one of the non-invasive methods that may have a potential role in the future diagnosis and screening of CRC. In addition, stage-specific expression of exosomal miRNAs might help establish a role for exosomal miRNAs in the staging, recurrence, and prognosis of CRC. Further experimentation is required before we recommend the use of such a molecular approach in controlling CRC.

## Materials and methods

### Ethical approval

The research experimental design conducted in this project followed the appropriate regulations and guidelines, including the Good Clinical Practice (GCP) Guidelines. The Biomedical Ethics Research Committee of King Abdulaziz University Hospital (KAUH), Jeddah, Saudi Arabia approved the study and assigned it registration number HA-02-J-008 with reference no. 510-20. All participants provided informed consent prior to their involvement. All blood samples were obtained at KAUH between years 2020 and 2021 after CRC patients as well as healthy individuals signed patient information and consent form. Participants were asked to complete a self-administered questionnaire regarding age, personal medical history and smoking. CRC was confirmed in patients based on monitored, diagnosed, and clinical data collected using standard clinical, endoscopic, radiographic, and histological criteria. Patients were qualified to participate in this study by surgeons and pathologists.

Inclusion criteria in the study healthy control arm was confirmed based on a recently completed normal colonoscopy with no previous history of CRC and no family history of cancer consistent with known dominant disorders. They also had no known genetic predisposition to the development of CRC. The exclusion criteria included CRC patients that have started chemotherapy or radiotherapy before blood collection and those identified to have hereditary non-polyposis CRC or familial adenomatous polyposis.

Samples were collected from CRC patients according to TNM staging system (American Joint Committee on Cancer AJCC). A total of 12 blood samples including nine CRC patients at stages II, III, and IV and three healthy individuals aged between 37 and 65 years old were utilized in this study. Samples were collected by a technician at endoscopy unit at KAUH. Clinicopathological characteristics of CRC patients are shown in Table 1.

**Table 1:** Clinicopathological characteristics of CRC patients. Tumor stages were detected according to staging system of AJCC, while location was detected according to proximal or distal classification.

Variable	Frequency (N = 12)
Gender	
Male	6
Female	6
Median range (37–65)	
Age at diagnosis	
TNM stage	
II	3
III	3
IV	3
Nodal stage	
Positive	5
Negative	4
Tumor location	
Rectum	3
Distal colon	4
Proximal colon	2

### Sample collection and preparation

Blood samples were collected and divided into serum collection tubes (Hebei Xinle Sci. & Tech. Co., LTD, China). Samples were left for 60 min at room temperature to fully coagulate, then centrifuged for 10 min at 3000 rpm (1900×*g*) and 4°C by using a swinging bucket rotor. Additional centrifugation was applied on serum samples after being transferred into 2 ml tubes. Serum samples were centrifuged for 15 min at 3000×*g* and 4°C to remove additional cellular nucleic acids attached to cell debris. Collected serum samples were aliquoted into 1.5 ml microcentrifuge tubes, then labeled and stored in −80°C refrigerator. All samples were processed within 3 h of collection.

### Exosome isolation

Exosomes were isolated using exoRNeasy Midi kit (Qiagen, cat. no. 77144) as described in manufacturer’s protocol except for adding buffer XE (45 ml) (Qiagen, cat. no.76214) instead of qiazol. Briefly, frozen serum (1 ml) was centrifuged for 5 min at 3000×*g* and 4°C after being completely thawed at room temperature to eliminate any remaining cell debris. Around 800 μl of clear supernatant was transferred to 2 ml new tube. By adding same volume of buffer XBP to sample and mixing gently. Mixture was added into exoEasy spin column and spun twice for 1 min at 500×*g* under cooling, and flow throw was discarded and then again spun for 1 min at 4000×*g* under cooling to remove liquid remaining on membrane. In this step, exosomes bound to exoEasy spin column. Around 3.5 ml washing buffer XWP was added onto spin column and centrifuged for 5 min at 4000×*g* under cooling. Then, spin column was transferred to a new collection tube. For eluting exosomes from membrane, 200 μl buffer XE was added and incubated for 1 min then centrifuged for 5 min at 500×*g* under cooling. For protein analysis, 1–2 ml buffer XE was added to membrane then ultrafiltration was applied to concentrate elution. To ensure all exosomes were collected, eluate was re-added onto exoEasy spin column and incubated for 1 min then centrifuged for 5 min at 5000×*g* under cooling.

### Transmission electron microscopy (TEM)

Examining exosomes by transmission electron microscopy (TEM) was done at King Abdullah University of Science and Technology (KAUST), Thuwal, Saudi Arabia. Purified exosomes (4 μl) were carefully loaded onto surface of pre-glow-discharged carbon-coated 300-mesh Cu formvar grid and incubated for 1 min for adsorption. Samples were then removed using filter paper and stained negatively for 1 min with 1 drop of 2% uranyl acetate. After removing stain with filter paper, sample was left to dry. Sample was then ready for examination under microscope. After loading samples on single tilt holder, they were inserted into Titan CT (FEI Company, Thermo Fisher Scientific). Finally, screening and imaging of exosomes were performed using several magnifications Gatan US4000 CCD camera (Gatan, Inc., Pleasanton, CA). ImageJ software was used to compute radius of exosomes based on images of exosomes obtained from TEM. Exosomes were then diluted in 1 mL PBS and size examined by Zetasizer Nano ZS (Malvern instruments) at 25°C according to manufacturer’s instructions.

### RNA extraction

For extraction of total RNAs, including miRNA, from exosomes and other extracellular vesicles (Evs), exoRNeasy Midi kit (Qiagen, cat. no. 77144) was used according to manufacturer’s protocol. To extract total RNA from vesicles, around 700 μl of lysate buffer ‘QIAzol’ was added into spin column and spun for 5 min at 4000 xg, then transferred to 1 ml tube. Lysate was incubated for 5 min at room temperature (15–25°C), which assists dissociation of nucleoprotein complexes. After incubation, 3.5 μl miRNeasy Serum/Plasma Spike-In Control was added as spike-in exogenous control and followed by adding 90 μl chloroform with shaking vigorously for 15 s to subsequent phase separation. Tube was incubated for 2–3 min at room temperature (15–25°C) and centrifuged for 15 min at 12,000 xg and 4°C. Through centrifugation, sample separates into 3 phases. An amount of 300 ul upper aqueous phase containing RNA was transferred to a new 2 ml collection tube. Then, two volumes of 100% ethanol were added and mixed thoroughly by pipetting slowly several times. Then, 650 μl of sample was transferred onto Rneasy MinElute spin column and centrifuged for 15 s. This step was repeated for remainder of sample. Then, 700 μl of buffer RWT was added to spin column and centrifuged for 15 s and 500 μl buffer RPE was added onto spin column and again centrifuged for 15 s. Then, 500 μl buffer RPE was added again onto spin column and centrifuged for 2 min. All centrifugations were done for each step at ≥ 8000×*g* (≥ 10,000 rpm) and in each time flow through was discarded except for last washing with buffer RPE. RNeasy MinElute spin column was transferred into a new 2 ml collection tube and centrifuged at high speed for 5 min with an open lid to dry membrane, then collection tube with flow through was discarded. Spin column was then transferred to a new 1.5 ml collection tube and 20–18 μl RNase-free water was directly added into center of membrane of spin column. Tube was incubated for 1 min and then centrifuged for 2 min with high speed to elute RNA. All processes of RNA extraction were done at room temperature (15–25°C) except phase separation process which was done at 4°C. Purified total RNA was measured by NanoDrop and Bioanalyzer, then frozen at −80°C until miRNA expression analysis was done.

### RNA quantification and qualification

Novogene Experimental Department has performed RNA quantification and qualification processes, and 1% agarose gels were used to monitor RNA breakdown and protein contamination. NanoPhotometer spectrophotometer was used to assess purity of RNA (Implen, CA, USA). RNA Nano 6000 Assay kit of Agilent Bioanalyzer 2100 system was used to test RNA integrity and quantity (Agilent Technologies, CA, USA).

### Small RNA library preparation and Illumina sequencing

Sequencing preparation was done by Novogene AIT Genomics (Singapore) utilizing NEBNext® Multiplex Small RNA Library Prep Set for Illumina® (NEB, United States). For miRNA library construction, 3 μg total RNA per sample was used as input material. NEBNext® Multiplex Small RNA Library Prep Set for Illumina®, NEB, USA) was used for generating sequencing libraries by following manufacturer’s recommendations. In summary, 3’ and 5’ adaptors were ligated to 3’ and 5’ ends of exososmal miRNA, respectively. Following hybridization with a reverse transcription primer, first strand cDNA was produced. PCR enrichment was used to create double-stranded cDNA library. Libraries containing insertions between 18 and 40 bp were ready for sequencing with SE50 after purification and size selection. Library was then tested for quantification using Qubit and real-time PCR, as well as size distribution detection using a Bioanalyzer 2100 system (Agilent Technologies, CA, USA). On Illumina platforms, quantified libraries were pooled and sequenced based on effective library concentration and data amount required.

### miRNA alignments and prediction of target genes

miRNAs were mapped to recognize known from novel miRNAs. To get candidate miRNAs and design secondary structures, we utilized miRBase20.0 as a reference, along with customized software mirdeep2^143^, and srna-tools-cli. Then, miRNA counts, and base bias were calculated using custom scripts on first location of identified miRNA with a certain length and on each position of all identified miRNA. Then, Rfam 12.0 database was applied to analyze clean reads matching miRNAs in addition to rRNAs, tRNAs, snRNAs, snoRNAs, and other non-coding RNAs. For identification of known and novel miRNAs, miRbase database (version 21.0) was utilized by mapping specific sequences of obtained reads to reference genome. Transcriptome sequencing results from genome were used as reference genome to perform target gene prediction. miRanda software was performed to predict target genes of known and novel miRNA^144^. Transcript per million (TPM) was measured to assess miRNA expression levels using equation Normalized expression = (mapped read-count/total reads) × 1,000,000)^25^.

### Differentially expressed exosomal miRNAs in CRC

Fold change (FC) values between CRC patients and healthy control were used to estimate miRNA differential expression. FC values were converted to base-2 logarithm of FC (log2 (FC)) once they were calculated. Exosomal miRNAs that were differentially expressed in dataset were then used to form Venn diagram and find miRNAs that showed statistical differences between CRC groups compared with healthy control. DESeq R package (1.8.3) was applied for differential expression analysis of two conditions or groups of biological replicates of samples. Benjamini and Hochberg method was used to adjust* P* values, while corrected* P* value of 0.05 was set as threshold for significant differential expression.

### Bioinformatic analysis

All predicted target gene of differentially expressed (DE) miRNAs were categorized into functional classes through Gene Ontology (GO) enrichment analysis by GOseq software based on Wallenius non-central hyper-geometric distribution^26^. All target genes that bind to DE miRNAs were applied in Shiny GO to identify KEGG pathways and GO enrichment terms FM, CC, and BP. Heatmap designed by iDEP.951 (http://bioinformatics.sdstate.edu/idep/) and KOBAS^145^ (http://kobas.cbi.pku.edu.cn/home.do) softwares was performed to test statistical enrichment of all DE miRNAs target gene candidates in KEGG pathways (http://www.genome.jp/kegg/). Binding sites of miRNAs on target gene was predicted using a built-in function of TargetScan database (v7.0; targetscan.org)^146^. Predicted target genes were visualized by Flourish studio (Flourish Studio, Kiln Enterprises Ltd, London, UK). Sequence of pre-miRNA stem-loop secondary structure was taken from miRBase and designed consulting ViennaRNA Web Services^147^.

### Verification of miRNA deep-sequencing results by qPCR

Freshly frozen serum exosomes were isolated to comprise the expression of miRNAs: miR-3184-5p and miR-423-5p at the qPCR level. Their respective target genes, *SMAD2* and *TGFBR2*, were selected to validate expression of these miRNAs (miR-3184-5p and miR-423-5p) resulted from deep sequencing. The *GAPDH* gene was used as a housekeeping gene. The primers used in the validation were designed on the basis of miRNA mature sequence (listed in Supplementary S8). cDNA synthesis and qRT-PCR were performed using StepOne Real-Time PCR System (Applied Biosystems, USA) according to the manufacturer’s protocol^148^. All samples were run in three replications. qPCR data were analyzed by two methods (i) ∆∆CT or Livak and (ii) ∆CT. The average CT for the endogenous control and each gene was calculated from the raw data generated by RT-PCR StepOne System and Data Assist software. The outliers were either omitted from the analysis or substituted with means. The graph Pad PRISM software and MS Excel functions were used to perform the analysis and generate the statistical plots of the data. The mean C_T_ and standard deviation for the reference gene (*GAPDH*), the two genes (*SMAD2* and *TGFER2*), and 2 miRNAs (miR-3184-5p and miR-423-5p) genes were calculated. P-value and fold change were calculated to determine the statistical significance of the expression pattern of miR-3184-5p and miR-423-5p genes (*P*-value < 0.05 and fold change >  ± 2).

### Supplementary Information

Supplementary Information 1.

Supplementary Information 2.

Supplementary Information 3.

Supplementary Information 4.

Supplementary Information 5.

Supplementary Information 6.

Supplementary Information 7.

Supplementary Information 8.

## Data Availability

Exosomal miRNA raw data are deposited in the European Nucleotide Archive at EMBL-EBI under accession number PRJEB64383. The datasets produced and/or examined in the course of this study are not publicly accessible because they are still undergoing further investigation. However, they can be obtained from the corresponding author upon a reasonable request.
