# Supplementary material for: Molecular detection of exosomal miRNAs of blood serum for prognosis of colorectal cancer
Source: Sci Rep. 2024 Apr 17;14:8902. doi: 10.1038/s41598-024-58536-3 (PMC11024162; doi:10.1038/s41598-024-58536-3)
Supplement: Supplementary file 8 — Supplementary Information 8. [file 41598_2024_58536_MOESM8_ESM.docx]

**Materials and methods of the real-time-PCR**

<https://www.mirbase.org/index.shtml>

<http://www.srnaprimerdb.com>

| **Gene Description** | Homo sapiens SMAD family member 2 (SMAD2), transcript variant 1, mRNA. |
| --- | --- |

| **Primer Pair Descriptions:** | | | | | | | |
| --- | --- | --- | --- | --- | --- | --- | --- |
| **Amplicon Size** | | 125 | |  | |  |  |
|  | | **Sequence** (5' −> 3') | | **Length** | | **Tm** | **Location** |
| **Forward Primer** | | CCGACACACCGAGATCCTAAC | | 21 | | 61.9 | 543-563 |
| **Reverse Primer** | | GAGGTGGCGTTTCTGGAATATAA | | 23 | | 60.1 | 667-645 |
| **Location in Coding Sequence** (primers and amplicon highlighted) | | | | | | | |
| 1 atgtcgtcca tcttgccatt cacgccgcca gttgtgaaga gactgctggg atggaagaag  61 tcagctggtg ggtctggagg agcaggcgga ggagagcaga atgggcagga agaaaagtgg  121 tgtgagaaag cagtgaaaag tctggtgaag aagctaaaga aaacaggacg attagatgag  181 cttgagaaag ccatcaccac tcaaaactgt aatactaaat gtgttaccat accaagcact  241 tgctctgaaa tttggggact gagtacacca aatacgatag atcagtggga tacaacaggc  301 ctttacagct tctctgaaca aaccaggtct cttgatggtc gtctccaggt atcccatcga  361 aaaggattgc cacatgttat atattgccga ttatggcgct ggcctgatct tcacagtcat  421 catgaactca aggcaattga aaactgcgaa tatgctttta atcttaaaaa ggatgaagta  481 tgtgtaaacc cttaccacta tcagagagtt gagacaccag ttttgcctcc agtattagtg  541 cc***ccgacaca ccgagatcct aac*agaactt ccgcctctgg atgactatac tcactccatt**  **601 ccagaaaaca ctaacttccc agcaggaatt gagccacaga gtaa*ttatat tccagaaacg***  ***661 ccacctc***ctg gatatatcag tgaagatgga gaaacaagtg accaacagtt gaatcaaagt  721 atggacacag gctctccagc agaactatct cctactactc tttcccctgt taatcatagc  781 ttggatttac agccagttac ttactcagaa cctgcatttt ggtgttcgat agcatattat  841 gaattaaatc agagggttgg agaaaccttc catgcatcac agccctcact cactgtagat  901 ggctttacag acccatcaaa ttcagagagg ttctgcttag gtttactctc caatgttaac  961 cgaaatgcca cggtagaaat gacaagaagg catataggaa gaggagtgcg cttatactac  1021 ataggtgggg aagtttttgc tgagtgccta agtgatagtg caatctttgt gcagagcccc  1081 aattgtaatc agagatatgg ctggcaccct gcaacagtgt gtaaaattcc accaggctgt  1141 aatctgaaga tcttcaacaa ccaggaattt gctgctcttc tggctcagtc tgttaatcag  1201 ggttttgaag ccgtctatca gctaactaga atgtgcacca taagaatgag ttttgtgaaa  1261 gggtggggag cagaataccg aaggcagacg gtaacaagta ctccttgctg gattgaactt  1321 catctgaatg gacctctaca gtggttggac aaagtattaa ctcagatggg atccccttca  1381 gtgcgttgct caagcatgtc ataa | | | | | | | |
| **GAPDH** | **TGTGGGCATCAATGGATTTGG** | | **ACACCATGTATTCCGGGTCAAT** | |  |  |  |

| **Gene Description** | Homo sapiens transforming growth factor, beta receptor II (70/80kDa) (TGFBR2), transcript variant 2, mRNA. | | | | | |
| --- | --- | --- | --- | --- | --- | --- |
| **Amplicon Size** | | 119 |  |  |  |  |
|  | | **Sequence** (5' −> 3') | **Length** | **Tm** | **Location** |  |
| **Forward Primer** | | AAGATGACCGCTCTGACATCA | 21 | 60.9 | 662-682 |  |
| **Reverse Primer** | | CTTATAGACCTCAGCAAAGCGAC | 23 | 60.7 | 780-758 |  |
| **Location in Coding Sequence** (primers and amplicon highlighted) | | | | | |  |
| 1 atgggtcggg ggctgctcag gggcctgtgg ccgctgcaca tcgtcctgtg gacgcgtatc  61 gccagcacga tcccaccgca cgttcagaag tcggttaata acgacatgat agtcactgac  121 aacaacggtg cagtcaagtt tccacaactg tgtaaatttt gtgatgtgag attttccacc  181 tgtgacaacc agaaatcctg catgagcaac tgcagcatca cctccatctg tgagaagcca  241 caggaagtct gtgtggctgt atggagaaag aatgacgaga acataacact agagacagtt  301 tgccatgacc ccaagctccc ctaccatgac tttattctgg aagatgctgc ttctccaaag  361 tgcattatga aggaaaaaaa aaagcctggt gagactttct tcatgtgttc ctgtagctct  421 gatgagtgca atgacaacat catcttctca gaagaatata acaccagcaa tcctgacttg  481 ttgctagtca tatttcaagt gacaggcatc agcctcctgc caccactggg agttgccata  541 tctgtcatca tcatcttcta ctgctaccgc gttaaccggc agcagaagct gagttcaacc  601 tgggaaaccg gcaagacgcg gaagctcatg gagttcagcg agcactgtgc catcatcctg  661 g***aagatgacc gctctgacat ca*gctccacg tgtgccaaca acatcaacca caacacagag**  **721 ctgctgccca ttgagctgga caccctggtg gggaaag*gtc gctttgctga ggtctataag***  781 gccaagctga agcagaacac ttcagagcag tttgagacag tggcagtcaa gatctttccc  841 tatgaggagt atgcctcttg gaagacagag aaggacatct tctcagacat caatctgaag  901 catgagaaca tactccagtt cctgacggct gaggagcgga agacggagtt ggggaaacaa  961 tactggctga tcaccgcctt ccacgccaag ggcaacctac aggagtacct gacgcggcat  1021 gtcatcagct gggaggacct gcgcaagctg ggcagctccc tcgcccgggg gattgctcac  1081 ctccacagtg atcacactcc atgtgggagg cccaagatgc ccatcgtgca cagggacctc  1141 aagagctcca atatcctcgt gaagaacgac ctaacctgct gcctgtgtga ctttgggctt  1201 tccctgcgtc tggaccctac tctgtctgtg gatgacctgg ctaacagtgg gcaggtggga  1261 actgcaagat acatggctcc agaagtccta gaatccagga tgaatttgga gaatgttgag  1321 tccttcaagc agaccgatgt ctactccatg gctctggtgc tctgggaaat gacatctcgc  1381 tgtaatgcag tgggagaagt aaaagattat gagcctccat ttggttccaa ggtgcgggag  1441 cacccctgtg tcgaaagcat gaaggacaac gtgttgagag atcgagggcg accagaaatt  1501 cccagcttct ggctcaacca ccagggcatc cagatggtgt gtgagacgtt gactgagtgc  1561 tgggaccacg acccagaggc ccgtctcaca gcccagtgtg tggcagaacg cttcagtgag  1621 ctggagcatc tggacaggct ctcggggagg agctgctcgg aggagaagat tcctgaagac  1681 ggctccctaa acactaccaa atag | | | | | |  |

Experient method One-step real-time RT-PCR

**hsa-miR-3184-5p UGAGGGGCCUCAGACCGAGCUUUU**

RP1(3184-5p) GGACGGTAGCAAGCAAAGAGTGTGAAAAGCTCGGT

RP2(3184-5p) GGGATTCTGGAAGATGATGATGACTGAGGGGCCTC

**hsa-miR-423-5p UGAGGGGCAGAGAGCGAGACUUU**

RP1(423-5p) GGACGGTAGCAAGCAAAGAGTGTGAAAGTCTCGCT

RP2(423-5p) GGGATTCTGGAAGATGATGATGACTGAGGGGCAGA

P1(universal) GGACGGTAGCAAGCAAAGAGTGTG

P2(universal) GGGATTCTGGAAGATGATGATGAC

sRNAPrimerDB: http://www.srnaprimer.org./

Cites: Xie et al., sRNAPrimerDB: a primer design and search tool for small non-coding RNAs

**Protocol for One-step real-time RT-PCR**

This protocol describes the detailed experimental procedure for one-step real-time RT-PCR using SYBR Green I. In the one-step approach, the entire reaction from cDNA synthesis to PCR amplification occurs in a single tube. You may need to modify this protocol if you use different reagents or instruments for real-time PCR.

1. **Introduction**

The principle of the one-step real-time RT-PCR is illustrated in Fig. 1. The whole detection procedure requires four primers: RP1, RP2, P1 and P2. RP1 contains the P1 and an 11-base sequence that is complimentary to the 3′-end of target miRNA. RP2 has P2 and a 11-base sequence that is same with the 5′-end of target miRNA. The total thermocycling program includes three stages. Stage 1 is the reverse transcription process. In this stage, the RP1 hybridizes with target miRNA, and then is extended in the presence of Reverse Transcriptase M-MLV (RNase H^-^) and dNTPs. Since the melting temperature (Tm) of an 11-base sequence is near 37 ℃, this stage is conducted at 37 ℃. In Stage 2, after denaturing at 95 ℃, the RP2 hybridizes with the cDNA of miRNA at 37 ℃, and both sequences are extended in the presence of hot-start Taq polymerase (HS Taq) and dNTPs at 60 ℃. Stage 3 is a conventional PCR process with the primers P1 and P2. The amplification of cDNA is monitored in the real-time PCR system using SYBR green I (Yan et al., 2013).


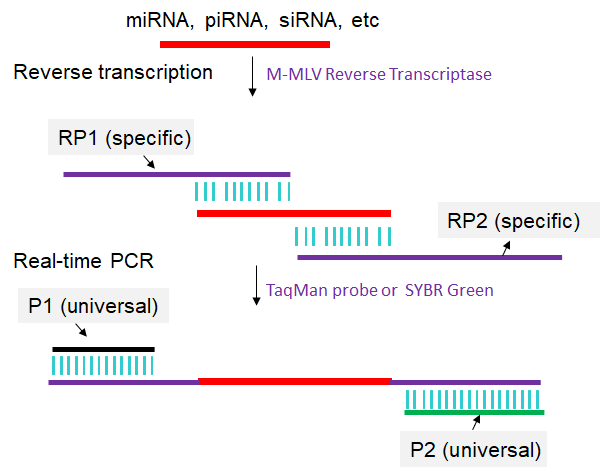


**Fig 1. Scheme for one-step real-time RT-PCR.**

For detailed experimental steps, please refer to the articles published by Yan et al., 2013.

**2. Material and Methods**

**2.1 Reagents and Equipment**

(1) Oligonucleotide Primers. miRNA specific primers designed from sRNAPrimerDB (<http://www.srnaprimerdb.com.>). These primers are ordered macrogen company). All the primers are desalted and both UV absorbance and capillary electrophoresis are used to assess the quality of primer synthesis.

(2) Total RNA, or miRNA.

(3) Optical tube and cap strips.

(4) M-MLV Reverse Transcriptase (RNase H^-^).

(5) SYBR Green PCR master mix.

(6) 50 bp DNA ladder.

(7) Quantitative PCR instrument.

(8) 3% agarose gel.

(9) Agarose gel electrophoresis apparatus.

**2.2 Procedure**

1. Prepare the following RNA/primer mixture in each tube. For each reaction:

| Total RNA | 0.5-1.0 μg |
| --- | --- |
| 5 X M-MLV Buffer* | 1 μL |
| 10 X PCR Buffer** | 1 μL |
| primer RP1 (2 μM) | 0.1 μL |
| primer RP2 (2 μM) | 0.1 μL |
| primer P1 (10 μM) | 0.1 μL |
| primer P2 (10 μM) | 0.1 μL |
| 20X SYBR Green I | 0.2 μL |
| Reverse Transcriptase M-MLV | 20 U |
| RNase inhibitor | 4 U |
| DEPC H_2_O | to 10 μU |

*5 X M-MLV Buffer (250 mM Tris-HCl, pH 8.3, 375 mM KCl, 15 mM MgCl_2_, 50 mM Dithiothreitol)

**10 X PCR Buffer (100 mM Tris-HCl, pH 8.3, 500 mM KCl, 15 mM MgCl_2_)

2. The real-time RT-PCR assay was conducted under the following conditions:

| Stage 1: | 45 ℃, 3 min; 37 ℃, 5 min, 1 cycle |
| --- | --- |
| Stage 2: | 95 ℃, 30 s; 37 ℃, 30s; 60 ℃ 30 s, 1 cycle |
| Stage 3: | 95 ℃, 30 s; 72 ℃, 30s, 40 cycles |

And the real-time fluorescence intensity was monitored at each cycle of the third stage. StepOne Real-Time PCR System (Applied Biosystems, USA) performed the reaction.

**2.3 Primers design**

The used example sequences of RNA and DNA oligonucleotides (5’ to 3’) are listed as follows:

**References:**

1. Yan J, Zhang N, Qi C, Liu X, Shangguan D. One-step real-time RT-PCR for detection of microRNAs. Talanta. 2013, 15;110:190-5. doi: 10.1016/j.talanta.2013.02.028.

**RT-qPCR Data Analysis**

**Aim of the study**:

To determine differentially expressed genes in patients compared to healthy control.

**Data:**

Mean threshold cycle (CT) values, derived from three replications of *SMAD2, TGFBR2*, 3184-5p, and 423-5p and *GAPDH* genes for cancer patients vs. healthy control, were used for analysis.

**Methodology:**

RT-qPCR data were analyzed by two methods (i) ∆∆C_T_ or Livak method and (ii) ∆C_T_ method. The average C_T_ for the endogenous control and each experimental gene was calculated from the raw data generated by RT-PCR StepOne System and Data Assist software. The outliers were either omitted from the analysis or substituted with means. The graph Pad PRISM software and MS Excel functions were used to perform the analysis and generate the statistical plots of the data.

1. **∆∆C_T_ or Livak method**

At first, we normalized C_T_ of the target gene to the C_T_ of the reference gene, then we normalized ∆C_T_ of the test sample to the ∆C_T_ of the control sample and finally we calculated the relative quantification (Rq) and differential expression (fold change, FC) using following steps:

1. Calculate ΔC_T_ values of each sample

ΔC_T_ for Ctrl = C_T_ target gene – C_T_ reference gene

ΔC_T_ for Test = C_T_ target gene – C_T_ reference gene

1. Calculate ΔΔC_T_ values for each comparison between two groups

ΔΔC_T_ = ΔC_T_ test sample – ΔC_T_ control sample

1. Calculate relative quantification (Rq) values for each comparison between groups:

Relative quantification (Rq) = 2 ^-∆∆CT^ = value*

* Rq >1 represents up-regulation whereas Rq <1 represents down regulation of gene

1. Calculate fold change (differential expression i.e how many times genes are up or down regulated?) for each comparison between groups, and fold change (FC) is derived from Rq: For Rq >1 (FC =Rq), for Rq=1 (FC=0) and for Rq<1 [FC = - (1/Rq)]
2. **∆C_T_ method (a variant of Livak method)**
3. Calculate ΔC_T_ values of each sample,

ΔC_T_ = [mean C_T_ reference gene – mean C_T_ target gene

1. Relative expression (RE) of each gene against each sample,

Relative expression of sample = 2 ^[ΔCT]^ = 2 ^[CT reference gene –CT target gene]^

1. Calculate the “ratio of relative expression” or relative quantification (Rq),

Ratio of relative expression (for control) = RE of control / RE of control =1

Ratio of relative expression (for test) = RE of test/RE of control = Rq*

*Rq >1 represents up-regulation whereas Rq <1 represents down regulation of gene

1. Calculate fold change (differential expression i.e how many times genes are up or down regulated?) for each comparison between groups, and fold change (FC) is derived from Rq: for Rq >1 (FC =Rq), for Rq=1 (FC=0) and for Rq<1 [FC = - (1/Rq)]

The Rq and FC either calculated by **∆∆C_T_ method** or **∆C_T_ method**, results in exactly same value. For more accuracy and reliability, we cross-checked the results using both methods.

**Statistical Analysis (t-test):**

The Rq values of the each gene was compared across all samples of two groups using one-tailed student’s t-test with unequal variance to calculate P-value for identification of significantly expressed genes.

**Result:**

We analyzed RT-qPCR data derived from a study conducted to determine differentially expressed genes in 8 patients compared to 3 healthy controls. The mean C_T_ and standard deviation for the reference (*GAPDH*) and 2 targets (*SMAD2, TGFBR2*) and 2 miRNAs (3184-5p and 423-5p) genes were calculated from the raw data (**Table 1**). We evaluated the expression pattern of SMAD2, TGFBR2, 3184-5p, 584-5p, 423-5p, 7a-5p, and miR-1843 genes for patients vs healthy control by both (i) ∆∆C_T_ (**Table 2**)

P-value and fold change was calculated to determine the statistical significance of the expression pattern of *SMAD2, TGFBR2*, 3184-5p, 423-5p genes (P-value <0.05 and fold change > ±2) (**Table 4**).

**Table 1:** Mean threshold cycle (mean C_T_) values of *SMAD2, TGFBR2*, 3184-5p, 423-5p and *GAPDH* genes in patients and healthy control were derived from experimental C_T_ values.

|  | **Genes (mean C_T_)** | | | | |
| --- | --- | --- | --- | --- | --- |
|  | SMAD2 | TGFBR2 | 3184-5p | 423-5p | GAPDH |
| S12 Stage IV | 20.42 | 21.91 | 21.48 | 22.40 | 18.10 |
| S11 Stage IV | 20.40 | 21.60 | 21.45 | 25.18 | 18.10 |
| S10 Stage IV | 20.42 | 21.56 | 21.48 | 25.18 | 18.10 |
| S9 Stage III | 22.34 | 19.61 | 24.49 | 25.98 | 20.73 |
| S8 Stage III | 22.34 | 19.61 | 24.49 | 25.98 | 20.73 |
| S7 Stage III | 22.34 | 19.61 | 24.49 | 25.98 | 20.73 |
| S6 Stage II | 19.80 | 19.10 | 19.30 | 19.40 | 19.53 |
| S5 Stage II | 19.80 | 19.10 | 19.30 | 19.40 | 19.53 |
| S4 Stage II | 19.80 | 19.10 | 19.30 | 19.40 | 19.53 |
| CONTROL | 27.67 | 25.21 | 27.66 | 27.90 | 25.47 |
| CONTROL | 27.67 | 25.21 | 27.66 | 27.90 | 25.47 |
| CONTROL | 27.67 | 25.21 | 27.66 | 27.90 | 25.47 |

**Table 2:** ΔC_T_ values of each sample/group was derived from their mean C_T_ values of target and reference genes, ΔC_T_ = [C_T_ target gene – C_T_ reference gene]

| **Groups** | **Genes (mean ΔC_T_)** | | | |
| --- | --- | --- | --- | --- |
|  | SMAD2 | TGFBR2 | 3184-5p | 423-5p |
| **ΔC_T_** **Healthy control** | 2.20 | 2.41 | 2.19 | 2.43 |
| **ΔC_T_** **Stage II** | 0.27 | -0.43 | -0.23 | -0.13 |
| **ΔC_T_** **Stage III** | 1.61 | -1.12 | 3.76 | 5.25 |
| **ΔC_T_** **Stage IV** | 2.32 | 3.46 | 3.38 | 7.08 |

**Table 3:** ΔΔC_T_ values as calculated by ΔΔC_T_ = [ΔC_T_ Test – ΔC_T_ Control]

| **Groups Comparisons** | **Genes (ΔΔCT)** | | | |
| --- | --- | --- | --- | --- |
|  | SMAD2 | TGFBR2 | 3184-5p | 423-5p |
| **ΔC_T_** **Stage II vs Healthy control** | -1.93 | -2.84 | -2.42 | -2.56 |
| **ΔC_T_** **Stage III vs Healthy control** | -0.59 | -3.53 | 1.57 | 2.82 |
| **ΔC_T_** **Stage IV vs Healthy control** | 0.12 | 1.05 | 1.19 | 4.65 |

**Figure 1:** Fold Change bar graph showing expression of *SMAD2, TGFBR2*, 3184-5p, 423-5p *genes*.
